# Molecular modelling and experimental validation of mangiferin and its related compounds as quorum sensing modulators of *Pseudomonas aeruginosa*

**DOI:** 10.1007/s00203-025-04240-3

**Published:** 2025-02-08

**Authors:** Nicolas Quinn Nortje, Jamiu Olaseni Aribisala, Charlene Pillay, Saheed Sabiu

**Affiliations:** Department of Biotechnology and Food Science, Faculty of Applied Sciences, Durban University of Technology, P.O. Box 1334, Durban, 4000 South Africa

**Keywords:** *Pseudomonas aeruginosa*, Biofilm, Quorum sensing, Mangiferin, Molecular dynamics simulation, Autoinducer

## Abstract

The LasR quorum sensing system regulates the virulence factors of *Pseudomonas aeruginosa*, a multi-drug resistant pathogen. Mangiferin and related compounds have been found to modulate this system as determined by in silico and in vitro experimental procedures. ZINCPharmer was used to compile a library of over 1000 metabolites that were screened to the top five based on shared pharmacophores and drug-like properties with mangiferin. Molecular docking and molecular dynamics simulation (140 ns) showed that ZINC E (− 55.64 ± 2.93 kcal/mol) and ZINC D (− 54.51 ± 2.82 kcal/mol) had significantly lower binding free energy compared to mangiferin-LasR (− 42.24 ± 3.94 kcal/mol) and the reference standard (azithromycin-LasR (− 40.01 ± 6.15 kcal/mol). ZINC D (95.16%) competed favorably with mangiferin (95.77%) as potential QS modulators at sub-minimum inhibitory concentrations relative to ZINC E (85.07%) and azithromycin (85.79%). These observations suggest mangiferin and related lead compounds as potential drug candidates for *P. aeruginosa* infection management.

**Supplementary Information:**

The online version contains supplementary material available at 10.1007/s00203-025-04240-3.

## Introduction

An opportunistic bacteria, *Pseudomonas aeruginosa* can infect major human organs and pose a serious risk to life, especially in immunocompromised patients (Cassin and Tseng 2019). *P. aeruginosa* often produces biofilms that function as a protective barrier, preventing the activities of antimicrobials, thus, resulting in multidrug resistance (MDR) to a variety of antimicrobial agents (Thi et al. 2020; Gebreyohannes et al. 2019). Furthermore, it has been shown that *P. aeruginosa* isolated from clinical samples have a biofilm-forming ability of 81.33% (Motevasel et al. 2024). Finding a novel solution to this problem is urgently needed, given the significance that biofilm development plays in the phenomenon of drug resistance in *P. aeruginosa* infections (Pattnaik et al. 2018).

Apart from biofilm generation, quorum sensing (QS) directs the activities of multiple *P. aeruginosa* virulence components, including alginate, aminopeptidase, chitinase, elastase, lipase, and protease, which facilitate microbial dissemination using the so-called autoinducer signaling molecules (Asfahl et al. 2022; O’Loughlin et al. 2013). Quorum sensing facilitates interspecies communication and crosstalk within a community by supporting cell communication (Asfahl et al. 2022). Four basic QS routes have been found in *P. aeruginosa*: the AI-based integrated quorum sensing system, RhlI/RhlR, Quinolone signal (PQS), and the LasI/LasR cascade (Asfahl et al. 2022; Pesci et al. 1997). All signaling systems work together to form an interconnected network that hierarchically regulates the production of virulence factors regulate by QS, even though each signaling system has an autoinducer that functions as a transcriptional activator and is activated when an internal concentration threshold is reached (Motevasel et al. 2024). At the highest level of the QS hierarchy is the LasI/LasR cascade, which regulates the operation of the other QS systems (Motevasel et al. 2024). A recent study observed a delay in QS responses in atypical strains of *P. aeruginosa* deficient in *LasR* (Soto-Aceves et al. 2021), an observation that demonstrates the importance of *LasR* in the activation and timing of QS. Thus, targeting the *LasR* receptors poses a novel mechanism of action to significantly impede the infectivity and overall pathogenicity of the *P. aeruginosa* (Naga et al. 2023; Zhao et al. 2020).

**Fig. 1:**
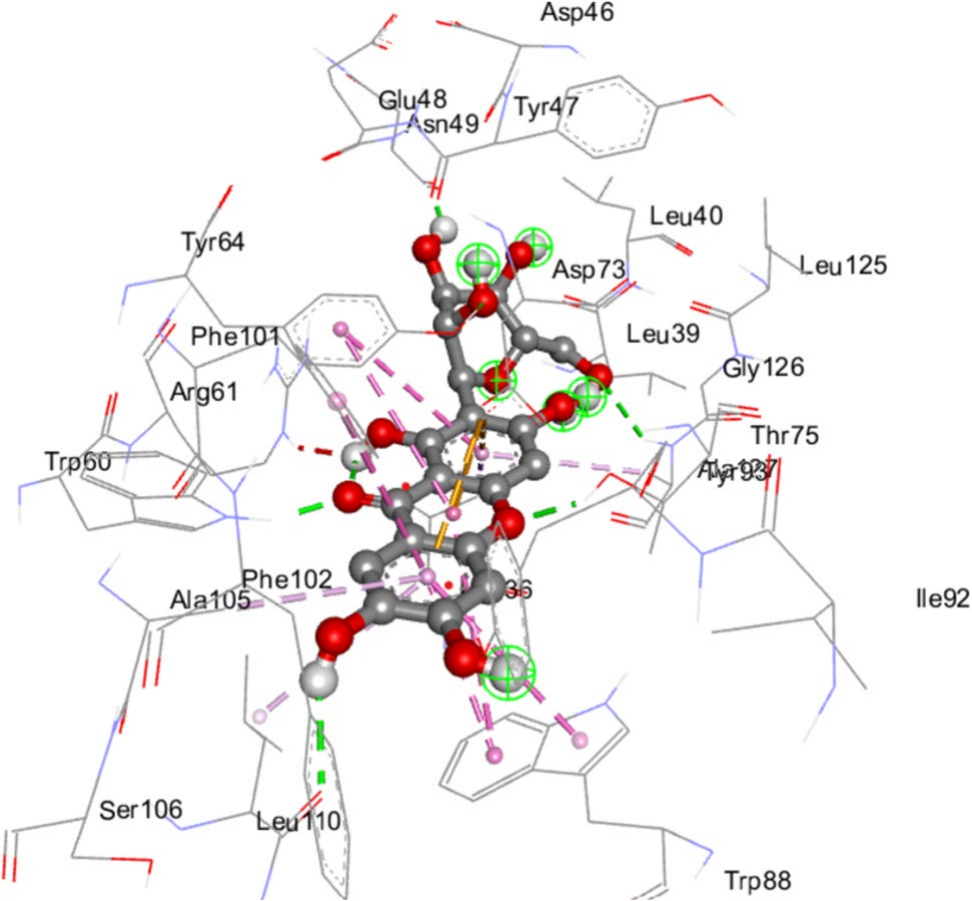
Interaction of mangiferin at the active site of LasR

With the reduced efficacy of the currently available conventional antibiotics due to biofilm formation and drug resistance, alternative sources of antibiofilm therapeutics have been investigated in plants and their metabolites (Gangwar et al. 2023; Teng et al. 2022). One such plant is *Mangifera indica* L. (mangoes) (Husain et al. 2017). Previous research has shown that the ethanolic extract of *Mangifera indica* has antibiofilm qualities against *Staphylococcus aureus* (Hepziba et al. 2023), and some oral bacterial pathogens (Soesanto et al. 2023). Specifically, Husain et al. (2017) showed that the aqueous and ethanolic leaf extract of *Mangifera indica* L. inhibited the QS system of *P. aeruginosa in vitro.* Although metabolites from *Mangifera indica* have been linked to the antibiofilm potential of its different extracts (Soesanto et al. 2023), the precise mechanism of action and primary metabolite(s) responsible for the observed effects remain elusive. However, a recent study has suggested that mangiferin, a polyphenolic compound of *Magnifera indica*, exhibits potent inhibitory effects on LasR to disrupt the QS mechanism in *P. aeruginosa* (Al-Shabib et al. 2024). It was proposed that mangiferin binds directly to LasR (Fig. 1), preventing it from forming a functional complex with 3-oxo-C12-HSL, thus inhibiting the activation of virulent genes. Hence in this study, the best chemically similar/related compounds to mangiferin were identified and validated in silico (using ligand-based pharmacophore, virtual screening, molecular dynamics (MD) simulation, and density functional theory (DFT) in addition to exploring its anti-QS effects. This was done to establish the antibiofilm potential of mangiferin while identifying other potent chemically related antibiofilm compounds with improved biological efficiency, reduced toxicity and favourable pharmacokinetics relative to mangiferin.

## Materials and methods

### Computational experimentation

#### Bioprospection of compounds similar to mangiferin

In accordance with Leach et al. (2010), compound bioprospection was performed. The structure data file (SDF) was retrieved from PubChem (https://pubchem.ncbi.nlm.nih.gov/) containing the 3D conformational structure for mangiferin and uploaded via the ‘’Load Features’’ tool of ZINCPharmer (http://zincpharmer.csb.pitt.edu/) (Koes and Camacho 2012). Once uploaded, a series of pharmacophores were displayed and gradually deselected as a screening tool. This prompted the “Submit Query” tool used to generate different compounds structurally similar to mangiferin based on selected pharmacophores, thereby establishing compounds related to mangiferin.

#### Molecular docking of identified compounds

The three-dimensional architecture of the target protein, LasR (PDB ID: 2UV0) was obtained from the online repository for the crystal structures of proteins, RCSB Protein Data Bank (https://rcsb.org/) (Bottomley et al. 2007), which was then optimized for docking readiness using UCSF Chimera v1. 15 (Petterson et al. 2004). The ligands, which included 133 bioprospected compounds in ZINCPharmer, mangiferin and the reference standard azithromycin, were optimized using the open babel program plug-in on Python Prescription (PyRx-0.8) 1.1.2 (Dallakayan and Olson 2015). The optimised protein and ligand were docked using the AutoDock Vina Wizard of the Python Prescription (PyRx-0.8) 1.1.2 (Dallakayan and Olson 2015). After docking, the complexes were sorted into the top twenty, including mangiferin, based on the docking scores closest to the native-ligand complex as a benchmark. The top twenty compounds including mangiferin and the reference standard were evaluated using GALAXY Europe (https://usegalaxy.eu/) to molecularly fingerprint each compound and generate a similarity matrix (Aribisala et al. 2021; Chiorcea-Paquim et al. 2020).

#### Pharmacokinetic properties of the top twenty compounds

The 20 lead compounds possessing the most negative docking scores were further screened to top five and the respective simplified molecular input line entry system (SMILES) from ZINCPharmer was evaluated using SwissADME (http://www.swissadme.ch/). The results were used to predict the compounds’ suitability as candidate drugs with reference to their absorption, distribution, metabolism, and excretion properties (Daina et al. 2017).

#### Molecular dynamic simulation of the top five compounds

The MD simulation was conducted utilizing the Graphical Processing Unit (GPU) of the AMBER 18 software package through an in-house program (HEAL1361) at the Center for High Performance and Computing (CHPC) located in Cape Town, South Africa (Case et al. 2005). The protein-ligand complex files obtained through molecular docking served as templates for the MD simulations. The compounds of interest or *LasR-*ligand systems were defined using the AMBER force field (FF18SB). As per Gonnet (2007), the adding of hydrogen atoms as well as the counter ions, Na^+^ and Cl^−^, ensured all systems were neutralized by the Leap AMBER 18 module. The sequence length for *LasR* is 175 and each ligand was assigned atomic partial charges by ANTECHAMBER. All non-bond interactions were adjusted by ANTECHAMBER to 8 Å. A 2000-step minimization was initialized, with constraints applied at 500 kcal/mol for an additional 1000 steps. This preceded another stage of minimization executed over 1000 unrestricted steps which used the conjugate gradient technique. The solutes were heated for 50 ps from absolute zero (0 K) to 300 K, maintaining a fixed volume of water and atoms, before being subjected to a collision frequency of 1.0 ps. Following that, each system was equilibrated for approximately 500 ps while the operating temperature remained constant at 300 K and restriction of hydrogen bonds was done using the SHAKE method. To further model an isobaric-isothermal ensemble (NPT), the system was subjected to randomized seeding, a pressure-coupling constant of 2 ps, a constant pressure of 1 bar as well as a Langevin collision frequency of 1.0 ps (Gonnet 2007). Each simulation employed the LEaP Module’s SHAKE algorithm which constrained expanding, bonds involving hydrogen through the SPFP precision model.

The co-ordinates and trajectories of the resulting systems were saved before they were analyzed as post-dynamic data [root-mean square deviation, root-mean square fluctuation and radius of gyration (RMSD, RMSF and RoG respectively)], achieved with the CPPTRAJ module. These trajectories were saved and visualized by plots generated using Origin V6 software (Seifert 2014). The binding free energy (ΔG) held within the individual *LasR-*ligand complexes was determined using the molecular mechanics/GB Surface Area (MM/GBSA) technique, over 140 000 extracted snapshots throughout the 140 ns trajectory. Of the 5 lead compound systems, the 3 lead compounds, as determined by those with the greatest binding free energy values, were selected for investigation of their electronic properties and further in vitro validation.

#### Density functional theory (DFT)

Calculations for density functional theory predicted the electronic characteristics of the top three lead compounds, using the DFT/B3LYP/631G/+ basic set of the Gaussian 16 program package of the CHPC, Cape Town, South Africa (Kruse et al. 2012; Parr et al. 1979). Electronic predictors such as chemical hardness, chemical softness, frontier orbitals, and energy gap were subsequently viewed by GaussView 6 software V.6.0.16 and visualized the frontier orbital characteristics of the 3 compounds as the lowest unoccupied molecular orbital (LUMO) along with the highest occupied molecular orbital (HOMO) (Almutairi et al. 2018).

### In vitro experimentation

#### Bacterial culturing and chemicals

The American Type Culture Collection (ATCC) strains of *P. aeruginosa* ATCC 27,853 and *C. violaceum* ATCC 12,472 were used in this study. These underwent repeated subculturing using Mueller-Hinton (MH) media for *P. aeruginosa* and Luria-Bertani (LB) media for *C. violaceum. P. aeruginosa* was incubated at 37 °C and *C. violaceum* in darkness under light-sensitive conditions at 30 °C. Azithromycin, used as the reference standard (Bala et al. 2011) and mangiferin obtained from Merck Sigma-Aldrich, Johannesburg, South Africa as well as the other two lead compounds (ZINC E and ZINC D) that were custom-made, were prepared using dimethyl sulfoxide (DMSO). *C. violaceum* was used as an indicator organism to visualize the production of violacein, a pigment controlled by *LasR*.

#### Antibacterial susceptibility testing of mangiferin, ZINC D and ZINC E

Tests for antibacterial susceptibility were conducted using the method of agar well diffusion (Balouri et al. 2016). Briefly, the bacterial strains of both *P. aeruginosa* (ATCC 27853) and *C. violaceum* (ATCC 12472) underwent standardization to 0.5 MacFarland standard (OD₆₀₀ = 0.08–0.1), and 0.02 ml were inoculated onto prepared MH agar, and evenly spread across the plate. Thereafter, a cork-borer with a 5 mm diameter was used to aseptically punch holes into the agar. In these wells, 0.08 ml of the lead compounds (mangiferin, ZINC E, ZINC D) (starting at 80 mg/ml), as well as the positive control (azithromycin: 2 mg/ml) and negative control (99% DMSO), were inoculated and left to incubate at 37 °C for *P. aeruginosa* ATCC 27,853 and 30 °C for *C. violaceum* ATCC 12,472 for 24 h. Thereafter, the zones of inhibition that indicated representing bacterial susceptibility to the respective treatment were observed (Bonev et al. 2008).

#### Minimum inhibitory concentration (MIC)

Sterile 96-well microtiter plates (Merck Sigma-Aldrich, Johannesburg, South Africa) were used to evaluate the minimum inhibitory concentration for each strain by following the broth microdilution method (Tenover 2009). MH broth (0.1 ml), 0.1 ml of standardized bacterial solution and 0.1 ml of the chosen test compound were added to each well. The added compound was serially diluted two-fold. The same was applied to the reference standard.

This process was repeated for *C. violaceum*, using only LB broth in place of MH broth. The experiment was conducted in three replicates which were maintained at optimal conditions for 24 h. Thereafter, 0.04 ml of p-iodonitrotetrazolium at a concentration of 0.2 mg/ml was introduced into each well with further incubation for 45 min. This allowed for colorimetric assay, visually comparing cell growth and inhibition, where wells that remained clear with no visible colour change were representative of successful inhibition, as opposed to those which turned pink (Eloff 1998). The lowest concentration to inhibit growth was recorded for subsequent testing.

#### Anti-quorum sensing assays

##### Qualitative anti-quorum sensing assay

For the qualitative AQS assay, *C. violaceum* was incubated for 24 h at 30 °C in sterile LB broth. The concentration of bacterial suspension was set to 0.5 MacFarland standard (OD₆₀₀ = 0.08–0.1) and evenly spread across sterile LB agar surfaces. Wells were punched into the plates and 0.1 ml of the control and 3 lead compounds in varying concentrations sub-MIC (azithromycin MIC: 0.125–1/8 MIC: 0.015 mg/ml; mangiferin MIC: 10 − 1/8 MIC: 0.625 mg/ml; ZINC E MIC: 10–1/8 MIC 0.625 mg/ml; ZINC D MIC: 10–1/8 MIC 0.625 mg/ml) were added. This was performed in triplicate with incubation for 24 h at 30 °C. As described by the Clinical and Laboratory Standards Institute M100-S17 (2007), QSI was determined by clear halos equivalent to 15 mm and greater, intermediate activity between 11 and 14 mm, and resistance to the compound shown by zones less than 10 mm.

##### Quantitative anti-quorum sensing assay

Quantification of the AQS effects of the top three lead compounds at sub-MIC concentrations, *C. violaceum* was carried out through a modified version of the spectrophotometric technique previously reported by Blosser and Gray (2000). A 96-well microtiter plate (Merck Sigma-Aldrich, Johannesburg, South Africa) was inoculated with 0.1 ml of standardized bacterial suspension along with 0.1 ml of the control and the 3 lead compounds (azithromycin MIC: 0.125–1/8 MIC: 0.015 mg/ml; mangiferin MIC: 10 − 1/8 MIC: 0.625 mg/ml; ZINC E MIC: 10–1/8 MIC 0.625 mg/ml; ZINC D MIC: 10–1/8 MIC 0.625 mg/ml). Thereafter, absorbance was measured for growth and violacein production at OD₆₀₀ and OD_485_ respectively. The plate was maintained at 30 °C for 24 h at 120 rpm, and the absorbance was measured again at OD_420_ before drying for a further 24 h. After drying, the contents of the wells were reconstituted by adding 0.15 ml of 100% DMSO. This mixture was then combined in a shaking incubator at 30 °C and 120 rpm for one hour. The absorbance at OD_485_ was then measured to ascertain the amount of violacein produced. This was determined across all three replicates, and the absorbance was obtained by calculating the mean. Thereafter, percentage inhibition was determined by employing the expression below.

$$\:Percentage\:inhibition\:\left( \% \right) = \frac{{{\text{OD}}600\:\:growth\:control - {\text{OD}}600\:\:test}}{{{\text{OD}}600\:\:growth\:control}} \times \:100$$

#### Cell attachment inhibition

The top three lead compounds (mangiferin, ZINC D, ZINC E) and the control azithromycin were further tested for AQS activity by a cell attachment inhibition assay modified from Sandasi et al. (2008). A 96-well microtiter plate was inoculated with 0.1 ml of 0.5 MacFarland standardized *P. aeruginosa* suspension, 0.1 ml of MH broth and 0.1 ml of the control and 3 lead compounds across different concentrations (azithromycin MIC: 0.125–1/8 MIC: 0.015 mg/ml; mangiferin MIC: 10 − 1/8 MIC: 0.625 mg/ml; ZINC E MIC: 10–1/8 MIC 0.625 mg/ml; ZINC D MIC: 10–1/8 MIC 0.625 mg/ml). A blank in the form of 0.2 ml MH broth accompanied the test compounds which were then left to incubate at 37 °C for 24 h.

Employing a slightly revised crystal-violet analysis method, planktonic growth and media were eliminated by washing the wells with sterile distilled water and blot-dried before being placed to air-dry in an oven overnight 30 °C to dry out remaining cells. Once dried, each well was reconstituted with 1% crystal violet (0.1 ml) to mark any persisting biofilm and incubated in the dark for approximately 15 min. Washing each well with sterile distilled water for another three times removes all unabsorbed staining, allowing semi-quantitative evaluation of biofilm formation to occur where the wells were de-stained with 0.125 ml of ethanol (95%), of which 0.1 ml was micropipetted into a new plate and read for absorbance at OD₅₈₅ (SpectraMax^®^ paradigm multimode microplate reader [Molecular Devices, Separations, South Africa]). In accordance with Sandasi et al. (2008), the inhibitory effects of the test compound as well as the controls were determined following criteria of ≥ 50% equating to high activity while values that fell in the range of 0 to 49% were considered low.

#### Biofilm development inhibition

##### Biofilm inhibition

The standardized bacterial suspension (0.1 ml) was micropipetted into a 96-well microtiter plate and maintained at 37 °C for 8 h before 0.1 ml of the test compounds and controls, in triplicates in the various investigated concentration ranges (MIC to 1/8 MIC) were transferred into each well. This was incubated for a further 24 h and stained as per the protocol described in “cell attachment inhibition assay”

##### Confocal laser scanning microscopy (CLSM)

The viability of the biofilm was assessed by applying confocal laser scanning microscopy (Quave et al. 2012), where the biofilm of *P. aeruginosa* was cultured on 1 × 1 cm coverslips, carefully inserted in 24-well polystyrene and placed in an incubator set at 37 °C for 8 h. Subsequently, the biofilm that was produced was further supplemented with the various concentrations of each test compound and controls in triplicate, before further incubation for 24 h. Deionized water was used to gently wash any adhered biofilm before staining with 0.01% acridine orange and kept incubating in total darkness for 3–5 min with a final washing before it was air dried. Fluorescence was detected using 488 nm excitation and a 500–530 bandpass filter and identified at x63 magnification employing a Zeiss LSM 710 (Carl Zeiss Microscopy, Jena, Germany) confocal laser-scanning microscope (Quave et al. 2012).

#### Inhibition of pyocyanin production

In accordance with Essar et al. (1990), an assay for pyocyanin production was conducted. Briefly, a 24 h culture of *P. aeruginosa* was standardized to 0.8 at OD₆₀₀ and micropipetted into centrifuge tubes with King’s A broth and the respective test compounds and controls at varied concentrations (MIC to 1/8 MIC). This was left to incubate overnight for 24 h at 37 °C before 1.5 ml of culture was transferred into Eppendorf tubes and centrifuged at 3000 x *g* for 10 min. Once completed, 1 ml of supernatant underwent brief chilling after transferal into ice-pre-cooled centrifuge tubes. Whilst still on ice, 0.1 ml of chloroform was added into each tube, as well as 0.2 M hydrochloric acid (0.3 ml) was added. This was mixed employing a vortex mixer (*Las*ec Vortex Genie 2, USA), and the separated layer which contained pyocyanin was read for absorbance at 520 nm in a 96-well microtiter plate. As this was done in triplicate, the mean OD value was taken and multiplied by the molar extinction coefficient (17.072) to determine the concentration of pyocyanin produced.

#### Motility assays: swarming and swimming

As there are two ways in which *P. aeruginosa* expresses *LasR* QS-associated motility (Li et al. 2014), assays for swarming and swimming (Yeung et al. 2009) were used to investigate the 3 lead compounds for their AQS activity by inhibition of QS-mediated motility.

##### Swarming motility assay

For the swarming assays, the medium required comprised of 3% glucose (*w/v*), 0.8% nutrient broth (*w/v*), and 0.5% agar (*w/v*) and left to dry once poured into plates. Standardized overnight culture of *P. aeruginosa* (2 µL of 0.5 MacFarland) was spotted onto the center of the swarming media before a further 2 µL of the control and 3 lead compounds azithromycin MIC: 0.125–1/8 MIC: 0.015 mg/ml; mangiferin MIC: 10 − 1/8 MIC: 0.625 mg/ml; ZINC E MIC: 10–1/8 MIC 0.625 mg/ml; ZINC D MIC: 10–1/8 MIC 0.625 mg/ml) were added (Köhler et al. 2000). Once the plates had incubated for 24 h, the diameters of the zones formed (mm) were assessed. This was carried out in three replicates and the mean zone size was calculated.

##### Swimming motility assay

The swimming assay followed that of Murray et al. (2010) with modifications including media prepared using 1% tryptone, 0.5% NaCl, and 0,5% agar, following inoculation and incubation as described by the procedure in “Inhibition of pyocyanin production assay”.

### Statistical analysis

The in silico and in vitro results unless otherwise indicated, were reported as the mean ± standard deviation and as percentages of inhibition (%). For the statistical studies, GraphPad Prism version 5.0 and one-way analysis of variance (non-parametric tests) were utilized to determine the significant difference (*p* < 0.05) between the treatment deviations.

## Results and discussion

### Computational experimentation

#### Molecular docking and related compound screening

A total of 133 unique hits (Table S1) were identified using the ligand-based pharmacophore where features inferring structural similarity to mangiferin were explored (Poola et al. 2023). Following molecular docking (Table S1), the reference standard (azithromycin) which was selected based on its established AQS by *LasR*-interference (Bala et al. 2011) performed poorly (−2.5 kcal/mol) compared to mangiferin and the related compounds (Table S1). While mangiferin had a good docking score of −10.2 kcal/mol against *LasR* consistent with what has been reported in a previous study by Meng et al. (2022), ZINC00192940 (−12.5 kcal/mol) had the lowest docking score among the related compounds suggestive of good binding capability with *LasR*. The high affinity of mangiferin reported in this study exceeded that reported in a separate study investigating the antimicrobial properties of mangiferin against *E. coli* LpxC (Prabhu et al. 2023).

Bond analysis of the docked complexes shows that there were 11 bonds in the *LasR-*azithromycin complex including a conventional hydrogen bond (Gln92), carbon-hydrogen bond (Hie72, Gln75), π-alkyl bond, van der Waals forces (Thr89, Pro68, Gln88, Ile86, Ile80, Ser76, Lys91, Ser71) (Table S2). The *LasR-*mangiferin complex had a total of 25 bonds comprising of a conventional hydrogen bond (Gly32), carbon-hydrogen bonds (Leu33, Gly120, Asp59, Trp54), π–π stacked (Tyr58), π–alkyl (Leu30, Val70, Ala64) and van der Waals forces (Arg65, Tyr41, Leu34, Ala44, Phe45, Leu119, Ile46, Arg55, Ala99, Leu104, Tyr50, Trp82, Asp67, Ser123, Thr109, Ala121). The *LasR-*ZINC A complex had a total of 22 interactions including π-sigma bonds (Leu30), π- π T-shaped (Tyr50), π-alkyl (Trp82, Ala121) and van der Waals forces (Trp54, Leu104, Tyr58, Ile46, Gly32, Ala44, Tyr41, Gly120, Leu34, Phe45, Leu119, Leu33, Val70, Ser123, Asp67, Phe95, Thr109, Thr69) (Table S2). The *LasR-*ZINC B complex showed 22 bonds including a conventional hydrogen bond (Thr109), attractive charge, salt bridge and π-anion (Asp67), carbon hydrogen bond (Gly120), π-alkyl, alkyl (Val70, Ala44, Leu34, Tyr41, Leu119, Ala121), π- π stacked (Tyr87) and van der Waals forces (Thr69, Trp82, Tyr63, Trp54, Tyr58, Leu30, Ile46, Gly32, Leu33, Thr74, Cys73, Ser123). The *LasR-*ZINC C complex showed 21 bonds including a conventional hydrogen bond (Asp67), unfavourable donor-donor (Tyr58), π-π stacked (Trp82), alkyl (Arg55, Ile46, Tyr41), π-alkyl (Leu30, Ala121) and van der Waals forces (Cys73, Val70, Ala54, Thr109, Ser123, Thr69, Trp54, Tyr50, Leu104, Ala99, Tyr87, Phe95, Pro68) (Table S2). The *LasR-*ZINC D showed 21 interactions, including two conventional hydrogen bonds (Asp67, Tyr58), a carbon-hydrogen bond (Ser123), π- π stacked and π- π T-shaped (Tyr41, Trp82), π-alkyl (Leu30, Ala121, Ala99, Val70) and van der Waals forces (Ala64, Ile46, Gly32, Phe31, Leu122, Leu104, Thr109, Phe96, Ile86, Pro68, Phe95, Thr69). The *LasR-*ZINC E showed the most number of interactions at 27 including one conventional hydrogen bond (Ser123), a salt bridge showed the most amount of bonds, totaling 27 including a conventional hydrogen bond (Ser123), a salt bridge, attractive charge and π-anion charge (Asp67), carbon-hydrogen bond (Leu104), unfavourable donor-donor bond (Tyr58), π- π T-shaped (Trp82), alkyl (Ala121), π-alkyl (Ala99, Phe96, Ile46, Leu30, Val70, Cys73, Tyr41, Leu119, Leu32, Ala121) and van der Waals forces (Ser100, Tyr87, Thr69, Thr109, Gly120, Leu33, Gly32, Ala33, Phe31, Arg55, Trp54). Surprisingly, *LasR-*ZINC D had the highest prevalence of hydrogen bonds which would ordinarily be the reason for having a higher docking score than *LasR-*mangiferin (Medeiros-Silva et al. 2017), however, *LasR-*ZINC E had the highest docking score and contradicted this observation. This could be as a result of greater diversity of interactions including van der Waals forces (Yang et al. 2023), incurring additional binding free energy in LasR-ZINC D complex.

Molecular docking provides a preliminary indication of ligand best fit on a protein with no consideration on dynamics and stability of the complex (Pérez and Tvaroška 2014), therefore, further energy refinement and dynamic research were conducted in this study.

**Fig. 2:**
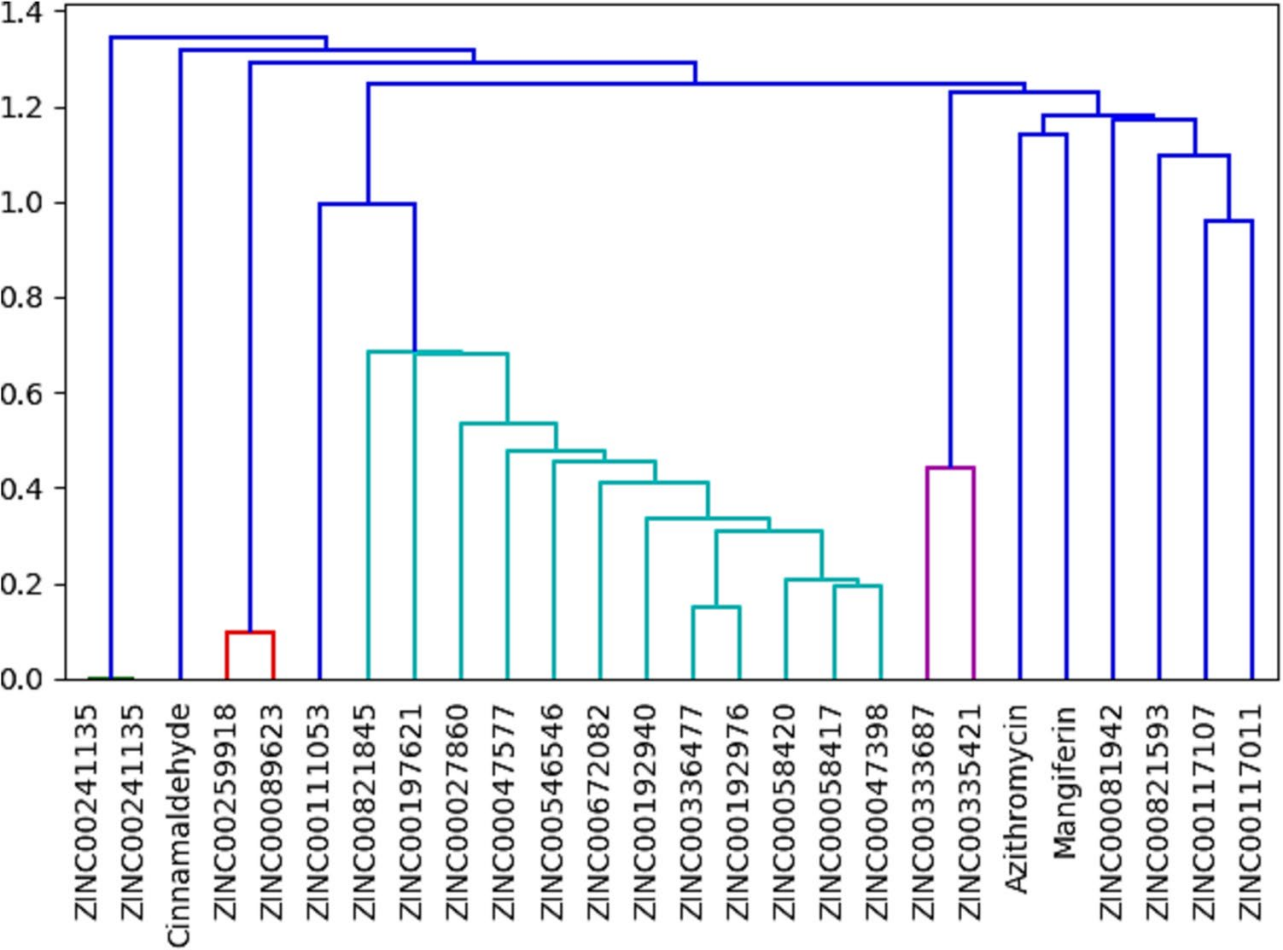
Molecular fingerprint of the top twenty bioprospected compounds with the closest chemical relatedness to mangiferin

The GALAXY Europe molecular fingerprinting (Fig. 2) identified that the screened compounds are structurally related and the six most closely related compounds amongst the top twenty highest scoring compounds with mangiferin are azithromycin, ZINC00117011 (ZINC A), ZINC00117107 (ZINC B), ZINC00333687 (ZINC C), ZINC00335421 (ZINC D) and ZINC00821593 (ZINC E) as shown by their similar colour (Fig. 2). This observation further affirmed the success and the efficacy of the ligand-based pharmacophore explored in this study in screening related compounds to mangiferin.

#### Pharmacokinetic properties of mangiferin and the top five lead compounds

An evaluation of the compounds’ pharmacokinetic properties guided the determination of their suitability for use as oral drugs. This process is typically conducted in drug discovery studies to predict the physicochemical characteristics of compounds regarding absorption, distribution, metabolism, excretion, and Lipinski’s Rule of five (Ro5). These parameters define a drug candidate as ideal for oral ingestion. A *Log*P value of below 5 is considered ideal for intestinal absorption (Sharma and Shanavas 2021).

Unlike the 5 lead compounds, it was found that mangiferin and the reference standard were the only molecules to violate Lipinski’s Ro5. Both exceeded the limit of number of hydrogen bond donors and acceptors (Table 1) whereas the 5 lead compounds were compliant.

Bioavailability represents absorption, with an ideal value considered to be 0.55 (Bojarska et al. 2020). Interestingly, mangiferin was below this value at 0.17, whereas the reference standard and 5 lead compounds were found to be 0.55 each (Table 1), suggesting mangiferin has poorer absorptive properties and might exhibit poor membrane permeability (Ma et al. 2014). High solubility and high gastrointestinal tract (GIT) absorption are considered ideal for oral drug candidacy, and mangiferin showed relatively poor permeability and low GIT absorption may be an indication that mangiferin might not demonstrate good oral bioavailability. This is because a larger dose would be required to compensate for the therapeutic delay in its distribution and absorption if drug development were to directly and solely follow Lipinski’s Ro5. Conversely, the 5 lead compounds had moderate-to-high solubility and high GIT absorption, suggestive of more efficient drug candidates than mangiferin. While this could imply lower doses of the 5 lead compounds requirement, it does not necessarily guarantee reduced toxicity or instability as medication (Kapalka 2009).

One potential advantage mangiferin has over two (ZINC C and ZINC E) of the five lead compounds would be no blood-brain barrier (BBB) permeance (Table 1). The BBB is responsible for the protection of the brain, preventing the inadvertent uptake of potentially neuroactive or toxic compounds (Pardridge 2012), so no permeation is considered ideal in drug discovery. However, this alone is not enough to disqualify ZINC C and E, as BBB permeance can be remedied following experimental modification and reengineering to optimize them for less invasive, more effective therapeutic delivery (Pardridge 2019). A lack of BBB permeance in mangiferin can be supported by another pharmacokinetic study investigating marine xanthone derivatives, which also did not exhibit BBB permeance (Loureiro et al. 2019). The significance of this lies in mangiferin being a xanthone glycoside, falling under the group of tested xanthones which as a family group could be subjected to testing for comparative or synergistic therapeutic effect.

**Table 1:** Molecular docking scores and pharmacokinetic properties of mangiferin and the top five related compounds against *LasR*

Compound	H-bond donors; acceptors	Lipinski violations	BBB permeant	Solubility	GIT absorption	Mass (g/mol)	Log *P*(0/w)	Bioavailability score	Docking score (kcal/mol)
Mangiferin	11; 8	2	No	Soluble	Low	422.34	0.71	0.17	− 10.2
Azithromycin	14; 5	2	No	Poorly soluble	Low	330.81	3.22	0.55	− 2.5
ZINC A (ZINC00117011)	6; 2	0	No	Poorly soluble	High	340.33	2.99	0.55	− 10.4
ZINC B (ZINC00117107)	3; 2	0	No	Moderately soluble	High	314.30	2.87	0.55	− 10.7
ZINC C (ZINC00333687)	6; 3	0	Yes	Moderately soluble	High	298.71	3.04	0.55	− 10.8
ZINC D (ZINC00335421)	3; 2	0	No	Moderately soluble	High	324.29	1.98	0.55	− 11.7
ZINC E (ZINC00821593)	3; 1	0	Yes	Moderately soluble	High	351.44	2.77	0.55	− 11.6

*BBB *Blood brain barrier, *GIT *Gastrointestinal tract, H-bond hydrogen bond, Log *P*(0/w) logarithm of the partition coefficient

#### Molecular dynamics simulations of mangiferin and the top five related compounds against *LasR*

The rationale behind MD simulations is to provide more accurate predictions of the behavioral patterns of atoms within a molecular system. This includes the structural properties of ligand-protein interactions introduced in molecular docking (Hollingsworth 2018). A higher or more negative binding free energy is an index of higher bond strength and higher affinity of a compound towards the target protein (Hata et al. 2021). Of the results obtained, the top five related compounds and mangiferin had higher negative binding free energy than azithromycin, with ZINC E (− 55.64 ± 2.93 kcal/mol) and ZINC D (− 54.51 ± 2.82 kcal/mol) having the most significant binding free energy values than mangiferin (− 42.24 ± 3.94 kcal/mol) against *LasR* (Table 2). These values serve as preliminary representations of thermodynamically favorable complexes (Sabiu et al. 2021a, b), implying ZINCs E and D as thermodynamically favorable inhibitors of LasR due to their higher binding free energy. A comparison of the intermolecular forces within each complex can explain the reason for the respective binding free energy values of each complex.

**Table 2:** Thermodynamic profiles (kcal/mol) and bond analysis of mangiferin and the top five related compounds against *LasR* after 140 ns MD simulations

Ligands	ΔE_vdW_	ΔE_elec_	ΔG_gas_	ΔG_solv_	ΔG_bind_	Number of hydrogen bonds	Number of hydrophobic bonds
Azithromycin	− 32.96 ± 4.6	− 212.96 ± 20.33	− 259.50 ± 22.42	219.49 ± 19.35	− 40.01 ± 6.15	1	3
Mangiferin	− 54.03 ± 3.15	− 18.94 ± 8.83	− 72.98 ± 8.83	35.73 ± 7.02	− 42.24 ± 3.94	5	16
ZINC A	− 48.01 ± 3.65	− 15.22 ± 6.74	− 63.23 ± 8.95	29.93 ± 6.01	− 35.3 ± 4.23	0	18
ZINC B	− 45.21 ± 2.46	− 23.04 ± 5.23	− 68.26 ± 5.37	30.13 ± 3.36	− 40.12 ± 3.44	2	16
ZINC C	− 43.2 ± 3.32	− 9.06 ± 7.01	− 52.27 ± 6.07	18.61 ± 4.38	− 35.66 ± 3.66	1	13
ZINC D	− 49.16 ± 3.16	− 190.74 ± 8.72	− 239.90 ± 8.53	187.39 ± 7.90	− 54.51 ± 2.82	3	11
ZINC E	− 54.69 ± 2.95	− 187.79 ± 7.81	− 242.48 ± 7.91	188.81 ± 7.03	− 55.64 ± 2.93	1	18

ΔEvdW van der Waals energy, ΔGbind total binding free energy, ΔEgas gas phase free energy, ΔEelec electrostatic energy, ΔGsolv solvation free energy

**Fig. 3:**
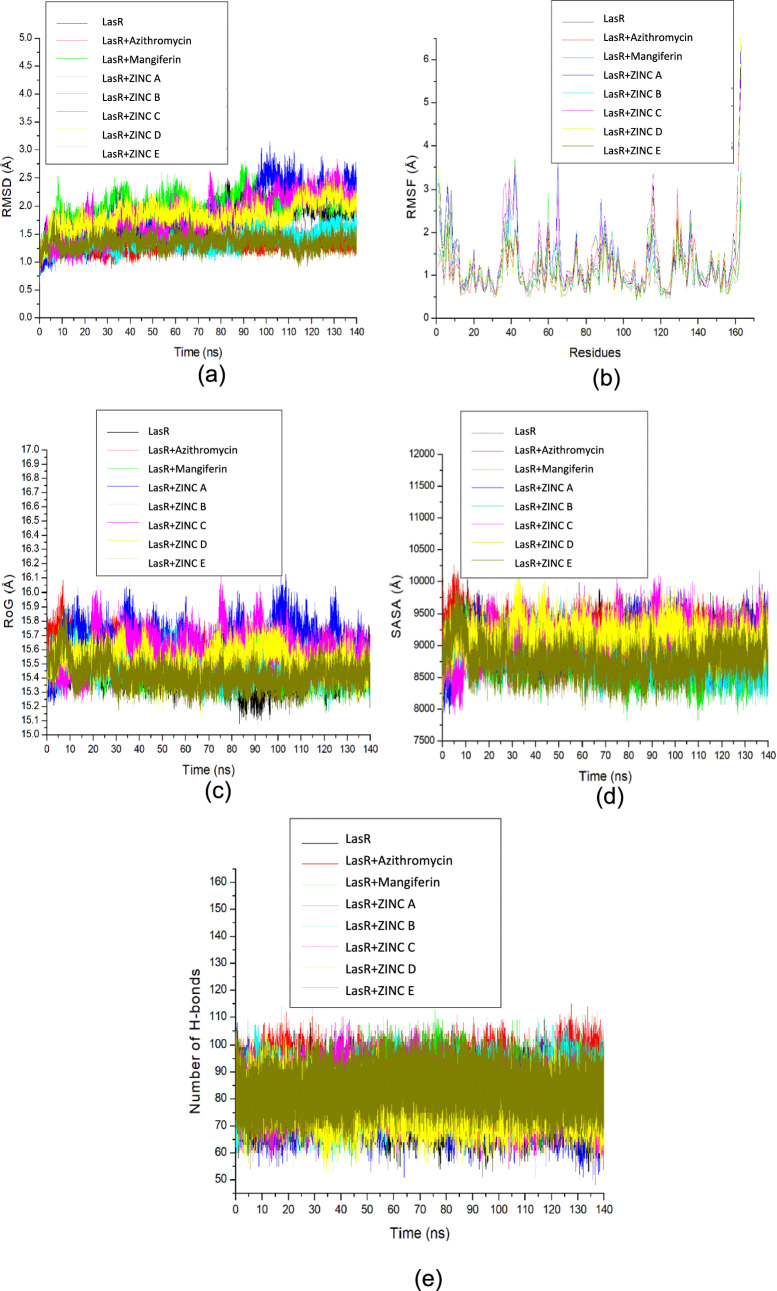
Comparative plots for **a** Root mean square deviation (RMSD) **b** root mean square fluctuation (RMSF) **c** radius of gyration (RoG) **d** solvent accessible surface area (SASA) and **e** number of intramolecular hydrogen bonds of apha carbon of *LasR*, mangiferin,  top five lead compounds and azithromycin complexes with *LasR* over 140 ns molecular dynamics simulation

Root mean square deviation (RMSD) aims to measure the configurational variations of mangiferin and the 5 lead compounds and reference standard once complexed with *LasR*. Typically, lower values reflect highly stable complexes and are visually represented by lower fluctuation peaks in stable complexes (Ramirez and Caballero 2016). Between 0 and 10 ns, the system was observed to be equilibrating with RMSD fluctuating between 0.75 Å and 1.75 Å. After 10 ns, each of the systems had an RMSD system that took a more diverse route which impacted their various average RMSD value (Fig. 3a). At 100 ns, *LasR-*ZINC A and *LasR-*ZINC C showed dynamic instability, deviating farther away from the apo-protein, with this conformational change indicating these complexes have not yet reached equilibration, unlike the other complexes. With the highest observed RMSD value being that of *LasR-*mangiferin (1.99 Å), the complexes fell within the acceptable 3 Å range suggested by Ramirez and Caballero (2016) for accurate docking reliability and stability representation.

Of the 5 lead complexes, the lowest RMSD value was that of *LasR-*ZINC E (1.35 ± 0.12 Å) which complemented the complex having the highest binding free energy (− 55.64 ± 2.93 kcal/mol) (Table 3). This suggests ZINC E has the highest binding affinity through its stability in complexing with *LasR*, and therefore the most promising AQS potential. ZINC B, E, and the reference standard complexes were also found to have the lowest RMSD values compared to the native protein structure (1.74 ±. 0.29 Å), whilst ZINC A, C, D, and mangiferin were shown to be higher. This observation suggests ZINC B, E, and the reference standard complexes displayed greater stability than ZINC A, C, D, and mangiferin in the inhibition of *LasR*.

Root mean square fluctuations (RMSF) represent amino acid fluctuations within the *LasR-*ligand complexes on account of the intra- and inter-molecular bonds formed. Lower fluctuation values and peaks reflect strengthened binding affinity between the ligand and protein (Rahimi et al. 2023) at the active amino sites. In this study, the lowest fluctuations observed close to the apo-protein were at amino residues 10–30 and 70–110 (Fig. 3b), by *LasR-*mangiferin, *LasR-*ZINC D, *LasR-*ZINC E, and the reference standard. This contradicted the observation made by Sharma et al. (2021) mentioning higher RMSF levels potentiating increased protein-ligand interactivity, as these ligand complexes showed the highest stabilities as per RMSD. Visible are ZINC A, B, and C as higher fluctuation peaks beyond the residue peaks of the apo-protein. This is suggestive of these three ligands causing greater deviation from the protein active sites rather than stabilizing with the catalytic residues, possibly due to higher flexibility or potential protein unfolding (Mali and Chaudhari 2018). This is especially noticeable in *LasR-*ZINC B (1.15 ± 0.51 Å) and *LasR-*ZINC C (1.34 ± 0.75 Å) (Table 3) which supports the lower binding affinities also reflected in RMSD. As such, these considerably weaker interactions at the binding site amino acids suggest *LasR-*ZINC A, *LasR-*ZINC B, and *LasR-*ZINC C to be suboptimal in terms of binding (De Vita et al. 2021) making them less promising *LasR* inhibitors.

Radius of gyration (RoG) is a thermodynamic measure of protein-ligand complex compactness, where lower values suggest much more compact and rigid complexes (Ghahremanian et al. 2022). Higher fluctuations are represented by higher values which represent reduced stability in the form of extended and potentially dynamic structures. This was repeatedly observed in *LasR-*ZINC A and *LasR-*ZINC C (Fig. 3c) from 80 to 120 ns. This reduced compactness could be attributed to increased flexibility as reflected in RMSF, but also protein unfolding or ligand unbinding from the protein (Zhu et al. 2017). *LasR-*mangiferin and *LasR-*ZINC E are comparable to the apo-protein, inferring stability from minimized protein perturbations (S’thebe et al. 2023) which supports their having higher stabilities and binding free energies than the other complexes.

An inspection of solvent accessible surface area (SASA) reveals the complexes’ interactions with solvent molecules. Higher exposure by water molecules represents reduced binding area available, mirrored by higher fluctuations as a complex experience increased structural changes such as unfolding whereas lower fluctuations imply a more compacted complex experiencing reduced solvent accessibility (Savojardo et al. 2021; Durham et al. 2009). *LasR-*ZINC E and *LasR-*mangiferin (Table 3) had the lowest SASA values compared to the reference standard and apo-protein suggesting the enhanced compactness of LasR following binding of ZINC and mangiferin (Fig. 3d).

The hydrogen bonds present within the compound-complexes were conducted at 50, 100, and 140 ns of the MD simulation (Fig. 3e). Hydrogen bonds contribute significantly to the stability of these complexes (Zikri et al. 2020). Overall, this was partially observed in this study’s findings which reflected the significance of bond prevalence when determining binding affinity.

At the start of the MD simulation, the highest number (27) of interactions and hydrogen bonds formed was recorded in *LasR-*mangiferin complex with 27 total bonds of which 5 were hydrogen bonds (Leu119, Gly120, Leu34, Arg55, Trp54) while the two complexes with the highest binding free energies were *LasR-*ZINC D had 28 including 4 hydrogen bonds (Tyr87, Asp67, Tyr50, Arg55) and *LasR-*ZINC E with 28 total bonds and 3 hydrogen interactions (Tyr58, Thr69, Ser129) (Table S2). This may coincide with the interactions formed by each complex by the end of the MD simulation, as the 3 lead compounds were revealed with the largest sum of total interactions as well as the prevalence of hydrogen bonds formed associated with higher binding affinities (Owoloye et al. 2022). These protein-ligand complexes may also have increased interactions at the active site (Pantsar and Poso 2018), which may further result in favourable binding within the protein-ligand, such as van der Waals forces (Yang et al. 2023) which contributes tremendously to bond-formation in drugs. *LasR-*azithromycin was revealed to have the lowest number of interactions throughout the simulation and a maximum of two hydrogen bonds (Gln92, Arg65) from 50 to 100 ns. *LasR-*ZINC A complex was consistently devoid of hydrogen bonds throughout the simulation, which would explain the high RMSD and RMSF values it held, including the lowest number of intramolecular bonds. The possible change in the number of intramolecular hydrogen bonds could be the result of bonds broken during the simulation and therefore equate to protein denaturation and structural deformation (Duan et al. 2009). This would have a significant effect on the bonds formed between the protein-ligand and how they would interact with each other. Based on RMSD, RMSF, RoG, SASA and number of intramolecular hydrogen bonds formed, the resulting binding free energies were used to choose 3 of the lead compounds for further in vitro evaluation: *LasR-*ZINC E (− 55.64 ± 2.93 kcal/mol), *LasR-*ZINC D (− 54.51 ± 2.82 kcal/mol) and *LasR-*mangiferin (− 42.24 ± 3.94 kcal/mol).

**Table 3:** Average root mean square deviation (RMSD), root mean square fluctuation (RMSF), and radius of gyration (RoG) values of the lead compounds and *LasR* complexes after 140 ns MD simulations

Ligands	RMSD (Å)	RMSF(Å)	ROG (Å)	SASA (Å)	Intramolecular H-bond
Apo *LasR*	1.74 ± 0.29	1.23 ± 0.71	15.44 ± 0.08	9028.96 ± 235	78.72 ± 6.5
Azithromycin	1.31 ± 0.10	1.08 ± 0.49	15.59 ± 0.09	9150.95 ± 222	86.50 ± 6.3
Mangiferin	1.99 ± 0.24	1.14 ± 0.58	15.45 ± 0.08	8748.43 ± 233	82.94 ± 6.4
ZINC A	1.84 ± 0.42	1.37 ± 0.78	15.64 ± 0.12	9033.89 ± 248	78.17 ± 6.5
ZINC B	1.41 ± 0.17	1.15 ± 0.51	15.47 ± 0.10	8827.07 ± 215	83.28 ± 6.1
ZINC C	1.81 ± 0.38	1.34 ± 0.75	15.60 ± 0.07	9112.56 ± 273	81.21 ± 6.6
ZINC D	1.83 ± 0.22	1.25 ± 0.74	15.51 ± 0.09	9133.83 ± 210	78.41 ± 6.4
ZINC E	1.35 ± 0.12	1.07 ± 041	15.41 ± 0.07	8761.06 ± 240	83.83 ± 6.3

*RSMD* Root mean square deviation, *RMSF* Root mean square fluctuations, *ROG* Radius of gyration,* SASA* Solvent accessible surface area

#### Density functional theory calculations of top three compounds

The top three compounds with the highest binding free energy underwent further probing for the electronic properties they each possessed to determine their stability and overall reactivity. This was done through DFT calculations to reveal potential descriptors (Table 4) such as the frontier orbitals: highest occupied molecular orbital (HOMO) and lowest unoccupied molecular orbital (LUMO). These are considered as significant identifiers of these compounds electronic properties as they describe the compound’s characteristics as an electron donor (HOMO) and electron acceptor (LUMO) (Alghuwainem et al. 2022). This information is useful in highlighting their reactivity as well as affinities for bond formation. The energy gap is provided once LUMO is subtracted from HOMO (Nagasundaram et al. 2022) and describes a compound’s stability in relation to its electronic properties. Low energy gaps reflect higher reactivity and a lower degree of stability, whilst the converse states higher energy gaps reflect lower reactivity and higher stability (Marinescu et al. 2020). This is the result of more energy required to excite electrons and cause electron transition from HOMO to LUMO (Kimber and Plasser 2023).

**Fig. 4:**
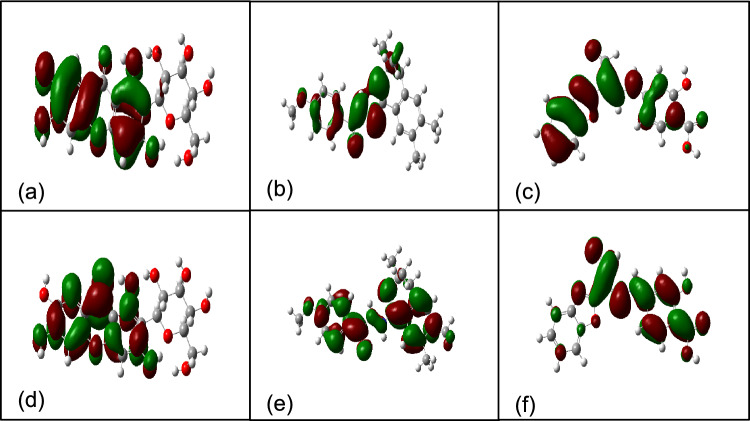
Visualizations of highest occupied molecular orbital (HOMO) in the upper row **a** mangiferin **b** ZINC D **c** ZINC D and lower row depicting lowest unoccupied molecular orbital visualization for **d** mangiferin **e** ZINC D **f** ZINC E

This relationship between energy gap and stability was predicted as findings show ZINC E had the highest energy gap (Table 4) as well as the highest binding free energy. ZINC D had the lowest energy gap, suggesting it to be the most reactive compound of the 3 lead compounds and thus might explain its better binding free energy relative to mangiferin in this study. The high reactivity associated with low energy gaps is the result of electronic charge transfers which leads to highly polarizable and thus highly reactive molecules (Nagasundaram et al. 2022). As ZINC D has the lowest energy gap of the 3 lead compounds, it is expected to be the most reactive, while the high LUMO value suggests a higher affinity towards electron-rich species such as the negatively charged residues of *LasR* (Tomaś et al. 2021). Mangiferin was found with the highest HOMO of the 3 lead compounds (Fig. 4a). This is suggestive of having the highest electron donation ability, hence a lower affinity for electron-rich species, and is supported by a separate study acknowledging the free-radical scavenging potential of mangiferin (Imran et al. 2017). On the other hand, ZINC D (Fig. 4e) had the highest LUMO to complement its higher reactivity.

Additionally, chemical hardness and chemical softness respectively reflect resistance and susceptibility to electron transfer. Higher chemical hardness is associated with higher energy gaps, hence improved compound stability and reduced reactivity (Jabbar 2021). This agrees with this study which found ZINC E to have the highest chemical hardness (Table 4). Overall, it could be gathered that due to a higher electronically defined reactivity, ZINC D might be the better inhibitor of QS as it would react with the *LasR* protein much better than the more stable ZINC E and mangiferin.

**Table 4:** Density functional theory-parameters of the top three compounds

	Mangiferin	ZINC D	ZINC E
LUMO E_LUMO_	− 2.32662409	− 2.36895614	− 1.02498183
HOMO E_HOMO_	− 6.31785363	− 6.01770574	− 5.33520385
Energy Gap Eg	3.99122954	3.6487496	4.31022202
Ionization Energy **(***I)*	2.32662409	2.36895614	1.02498183
Electron Affinity **(***A)*	6.31785363	6.01770574	5.33520385
Hardness (η)	1.99561477	1.8243748	2.15511101
Softness (*S*)	0.501098717	0.548132982	0.46401322
Electronegativity	5.485550905	5.37780901	4.822712935
Chemical Potential	− 5.485550905	− 5.37780901	− 4.822712935
Global electrophilicity	7.539348071	7.926230331	5.396139676

### In vitro evaluation

#### Minimum inhibitory concentration

To investigate the inhibitory effects of the top three lead compounds at sub-bactericidal concentrations, it was necessary to determine the MIC of each. This served as a starting point from which QSI could be investigated without affecting growth. A colorimetric assay followed the addition of iodonitrotetrazolium (INT), where colour changes were observed at lower concentrations indicating no inhibition, but rather proliferation. Higher concentrations were inspected for clear white zones which defined successful inhibition of growth. Of the 3 lead compounds, mangiferin had the highest MIC concentration (5 mg/ml), whilst ZINC E was on par with azithromycin (2.5 mg/ml), and ZINC D exhibited the lowest and thus strongest MIC (0.625 mg/ml) (Table 5). Mangiferin having the highest MIC is justified as phytocompounds are typically noted for requiring higher concentrations to inhibit bacterial growth compared to antibiotics (Kyaw and Lim 2012). However, the MIC range for a phytocompound to be classed as an antimicrobial is below 0.1 ml/ml (Taguri et al. 2006). Chemical optimization can be suggested in the context of synthesizing promising derivatives from lead compounds (Khameneh et al. 2021).

**Table 5:** Minimum inhibitory concentration (mg/ml) of the top three lead compounds and controls against *P. Aeruginosa*

Concentration	DMSO	Mangiferin	ZINC D	ZINC E	Azithromycin
MIC	Inactive	5.000	0.625	2.500	2.500
½ MIC	Inactive	2.500	0.312	1.250	1.250
¼ MIC	Inactive	1.250	0.156	0.625	0.625
1/8 MIC	Inactive	0.625	0.078	0.312	0.312

#### Anti-quorum sensing

##### Qualitative anti-quorum sensing assay

**Fig. 5:**
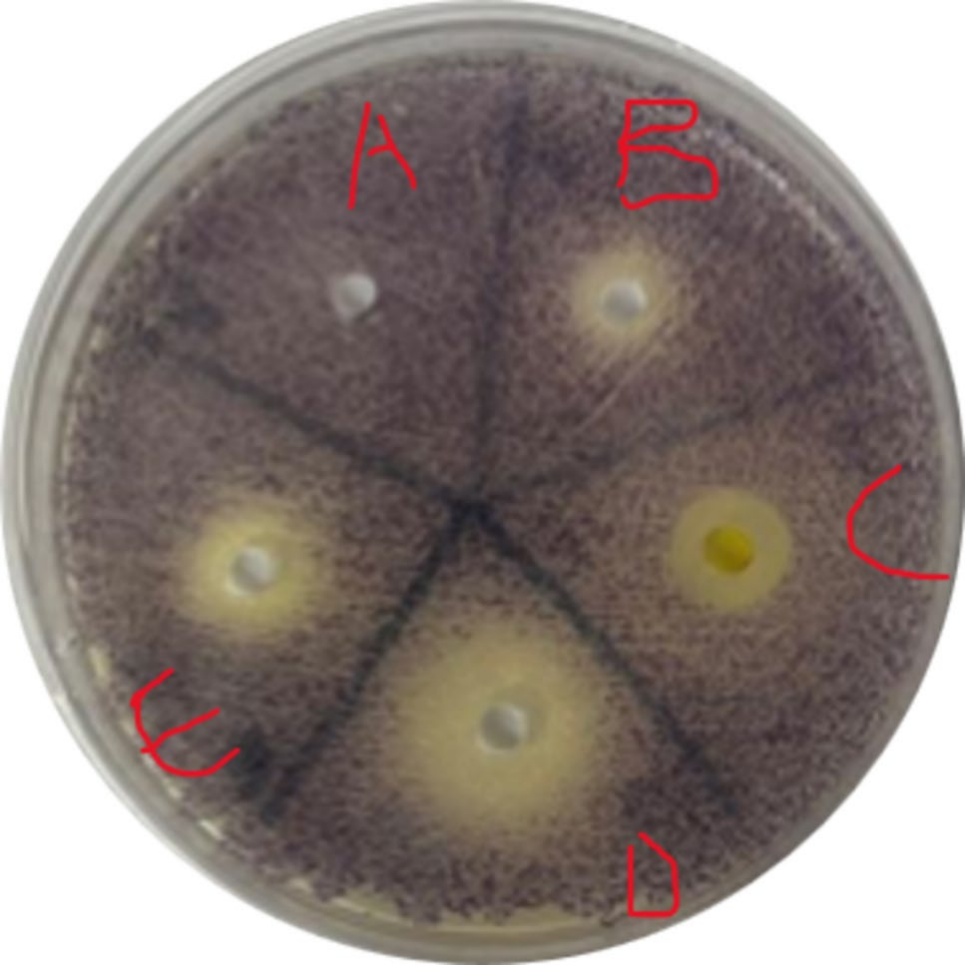
Violation inhibition zone of MIC concentrations of the test compounds. **A**: DMSO, **B**: Mangiferin, **C**: ZINCE, **D**: Azithromycin, and **E**: ZINCD

*C. violaceum* is renowned as a biomonitor for its QS-mediated production of the purple pigment violacein (Venkatramanan et al. 2020) and was exploited to visualize inhibition of the phenotype as AQS potential wielded by the test compounds at sub-MICs. The reference standard showed the highest degree of inhibition by exhibiting bacterial growth and halo clearing or opaque zones, compared to the negative control’s plate which appeared as a solid purple indicator of QS execution (Fig. 5).

This opaque zone, with visible cellular growth present, indicated successful QSI and was compared with the test compounds’ varying degrees of production inhibition. Mangiferin was found to show the largest zone at 20 mm, which exceeded the largest zone of inhibition obtained by Husain et al. (2017), albeit this was of *M. indica* extract in acetone, and at significantly lower concentrations. ½ MIC was the only concentration at which mangiferin was found to induce *C. violaceum* susceptibility. This is unlike ZINC D and E which, although presented smaller zones of violacein inhibition maintained consistently intermediate-to-susceptible AQS activity, with the zone size ranging from 10 to 17 mm (Table 6). Interestingly, ZINC D showed larger zones as concentration decreased, whereas ZINC E gradually increased zone size. While these results may not be comparable to studies with larger zones of inhibition achieved in antibiotic derivatives as mentioned by Naga et al. (2021), the findings obtained strongly affirm the preliminary in silico prediction of the test compounds binding to *LasR*. This is especially noticeable in ZINC D, as the in silico findings revealed that it binds to the target protein with the highest reactivity and affinity. This affinity could influence its specificity and contribute to the reason for its lower concentration showing increased inhibition, in conjunction with the MD simulation finding ZINC D to be closest in value to the native enzyme’s state.

**Table 6:** Violacein inhibition zone diameter (mm) of MIC and sub-MIC concentrations of the test compounds against *C. Violaceum*

Concentration	DMSO	Mangiferin (mm)	ZINC D (mm)	ZINC E (mm)	Azithromycin (mm)
MIC	Inactive	11.33 ± 0.47	15.33 ± 1.24	14.33 ± 0.47	28.75 ± 0.81
½ MIC	Inactive	20 ± 0.81	11.66 ± 0.81	17.00 ± 0.00	23 ± 1.63
¼ MIC	Inactive	10.33 ± 0.00	13.00 ± 0.00	15 ± 1.41	17 ± 1.63
1/8 MIC	Inactive	9.00 ± 0.00	10.33 ± 0.94	16.33 ± 0.81	13.00

##### Quantitative anti-quorum sensing assay

**Fig. 6:**
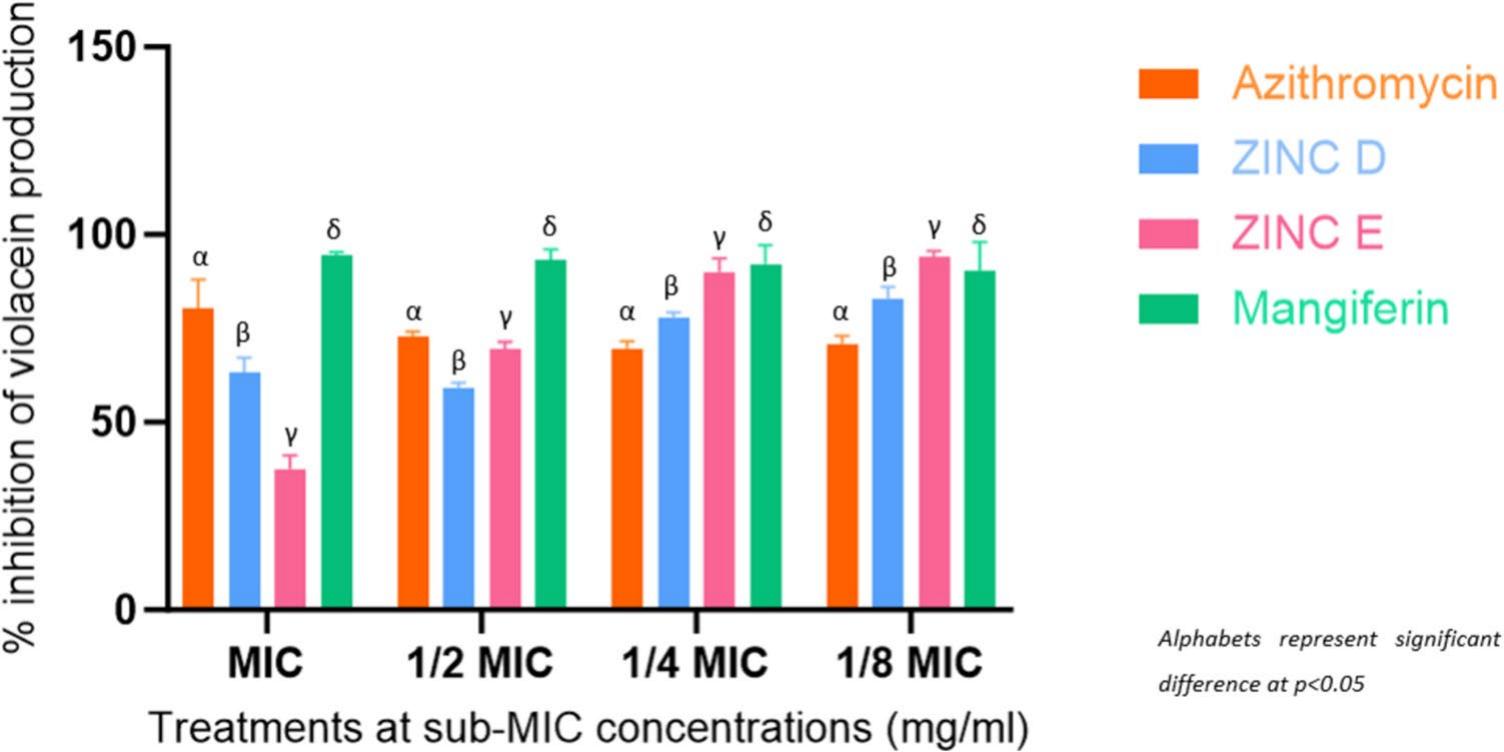
Quantitative assessment of inhibitory effects of 3 lead compounds and control on the violacein production by *C. violaceum* ATCC 12,472 at sub-MIC concentrations (mg/ml) of azithromycin at 0.25 − 0.015 mg/ml (MIC to 1/8 MIC), mangiferin 10–0.625 mg/ml (MIC to 1/8 MIC), ZINC E 10–0.625 mg/ml (MIC to 1/8 MIC), ZINC D 10–0.625 mg/ml (MIC to 1/8 MIC)

With promising QSI effects demonstrated in a qualitative assay, a quantitative analysis was necessary to ascertain the effectiveness of the 3 lead compounds. This was achieved by enumerating violacein production inhibition. This revealed a concentration-dependent effect for azithromycin, which would be typical of an antibiotic. Remarkably, mangiferin contradicted the results of the qualitative assay as it exhibited extremely high inhibition of violacein production at all tested concentrations. While this was unexpected, the findings are supported by those of Husain et al. (2017), who found *M. indica* phytoconstituents to inhibit violacein production by as much as 83.6%. Although this was found to be at lower concentrations, such high inhibition at low concentrations may be attributed to additional bioavailable compounds within their tested extracts, including bioavailable mangiferin. ZINC D (58.12 − 85.07%) and ZINC E (34.98 − 95.16%) (Fig. 6) both demonstrated AQS activity independent of concentration, as both exhibited progressively increasing percentage inhibition as concentration decreased. This could be the result of decreased doses bearing higher specificity when targeting the protein, potentially complementing the observation made of their similarities in structure to the native ligand conformer. This is in complete opposition to the reference standard, indicating to be more effective at lower and potentially less toxic concentrations than azithromycin. A similar study that explored *Passiflora edulis* extracts (Venkataramanan et al. 2020) corroborates the inhibition expressed by mangiferin, noting values as high as 75.8% inhibition which was attributed to AHL-inactivation by its extracts, rather than action by protein-inhibition as predicted in this study.

#### Cell attachment inhibition

**Fig. 7:**
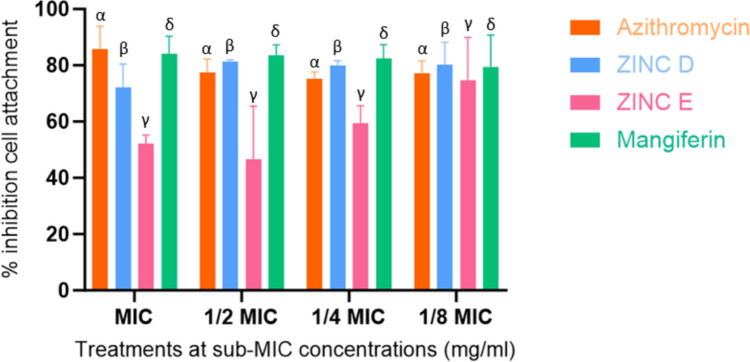
Inhibitory effect of the test compounds and controls on *P. aeruginosa* ATCC 27,853 cell attachment at sub-MIC concentrations (mg/ml) of azithromycin at 0.25 − 0.015 mg/ml (MIC to 1/8 MIC), mangiferin 10–0.625 mg/ml (MIC to 1/8 MIC), ZINC E 10–0.625 mg/ml (MIC to 1/8 MIC), ZINC D 10–0.625 mg/ml (MIC to 1/8 MIC)

As cell attachment is an effect of QS, an assay for such was carried out in *P. aeruginosa.* Cinnamic acid derivatives were previously communicated by both Paluch et al. (2020) and Wang et al. (2019) to inhibit cell attachment by inhibition of *LasR* and *Rhl*. Similarly, inhibition of *LasR* was demonstrated in this study, as cell attachment was successfully reduced. The control was revealed to initially have the highest percentage inhibition (73.67–83.4%) (Fig. 7), following Kumar et al. (2021) who reported azithromycin’s capacity to reduce extracellular matrices and subsequent adherence to surfaces, which resulted in reduced biofilm expression. Once again, a concentration-dependent effect was observed. Mangiferin showed the highest percentage inhibition (77.99–88.54%) of cell attachment, consistently maintained across all tested concentrations which reflects the results obtained in the qualitative AQS assay. However, test compounds ZINC D and E showed higher inhibition levels than expected. ZINC E demonstrated an effect independent from concentration, as lower concentrations visibly yielded higher percentage inhibition (Fig. 7), although it showed the least effect of the 3 lead compounds tested. ZINC D followed a similar pattern, as sub-MIC concentrations showed relatively high inhibition, more than the reference standard and on par with mangiferin. This astonishing level of inhibition agrees with Subramanian et al. (2022), wherein it was found that increased QSI resulted from sub-lethal or sub-bactericidal concentrations, otherwise allowing for ‘easier eradication’ (Qu et al. 2016). Thus, reduced biomass is available to facilitate the development of biofilm.

#### Biofilm development

##### Biofilm development inhibition

Biofilm has proven to be one of the most significant QS-mediated phenotypes, its production heavily contributing to the overall pathogenicity and longevity of infection (Lazar et al. 2021). Due to its ability to inherent ability to resist the uptake of material harmful to cells, targeting its development in the form of *LasR* in QS makes for a promising target. This was qualitatively affirmed in *P. aeruginosa* which is susceptible to QSI by the 3 lead compounds and was quantified. Surprisingly, the reference standard performed poorly in relation to the 3 lead compounds. At MIC, azithromycin was found to express its most effective inhibition. It contradicted not achieving 50% for it to be considered an effective enough treatment for *P. aeruginosa.* This contradicted notable antibiofilm activity as mentioned by Ding et al. (2018).

**Fig. 8:**
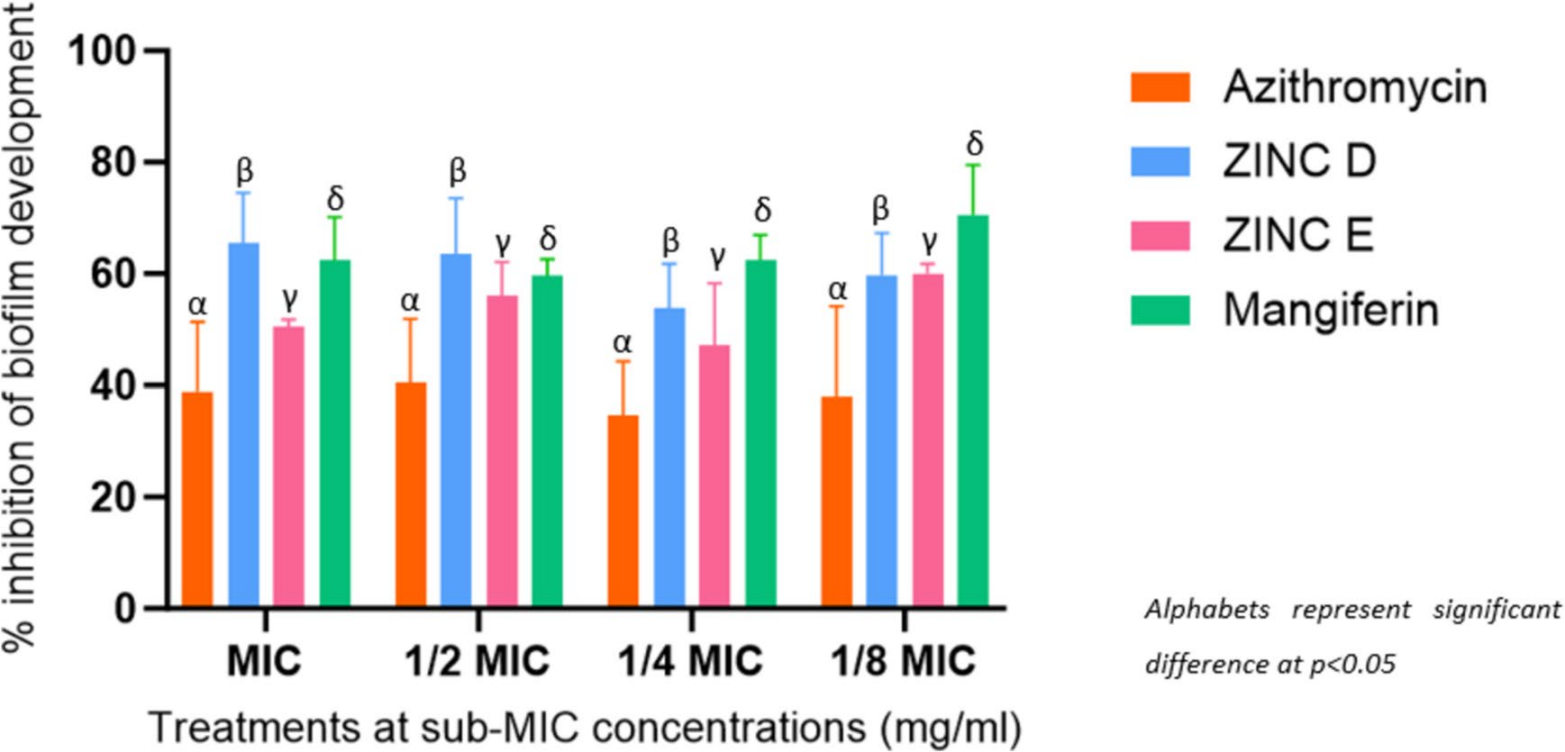
Inhibitory effect of the test compounds and controls on biofilm development of P. aeruginosa ATCC 27,853 at sub-MIC concentrations (mg/ml) of azithromycin at 0.25 − 0.015 mg/ml (MIC to 1/8 MIC), mangiferin 10–0.625 mg/ml (MIC to 1/8 MIC), ZINC E 10–0.625 mg/ml (MIC to 1/8 MIC), ZINC D 10–0.625 mg/ml (MIC to 1/8 MIC)

The same cannot be said for the 3 test compounds, as all 3 demonstrated significantly higher inhibition of biofilm formation than azithromycin. The highest percentage of inhibition was achieved by mangiferin (70.61%) at 1/8 MIC. This is a significant revelation, as the observed trend of higher inhibition at lower concentrations becomes increasingly apparent, particularly in ZINC D and ZINC E. The potential of mangiferin to inhibit biofilm development at all concentrations was also supported by Husain et al. (2017) who found *M. indica* extract to demonstrate biofilm reduction in both *P. aeruginosa* and *Aeromonas hydrophilia* (36 − 82%). While ZINC E visibly showed the least inhibition of the 3 lead compounds (Fig. 8), ZINC D showed the second highest, consistent inhibition alongside mangiferin, with greater inhibition exhibited at MIC and ½ MIC. However, all compounds excluding the reference standard were revealed to show greater than 50% inhibition of biofilm development in *P. aeruginosa* at 1/8 MIC, confirming not only adequate AQS activity but promisingly so at lower concentrations.

##### Confocal laser scanning microscopy

**Fig. 9:**
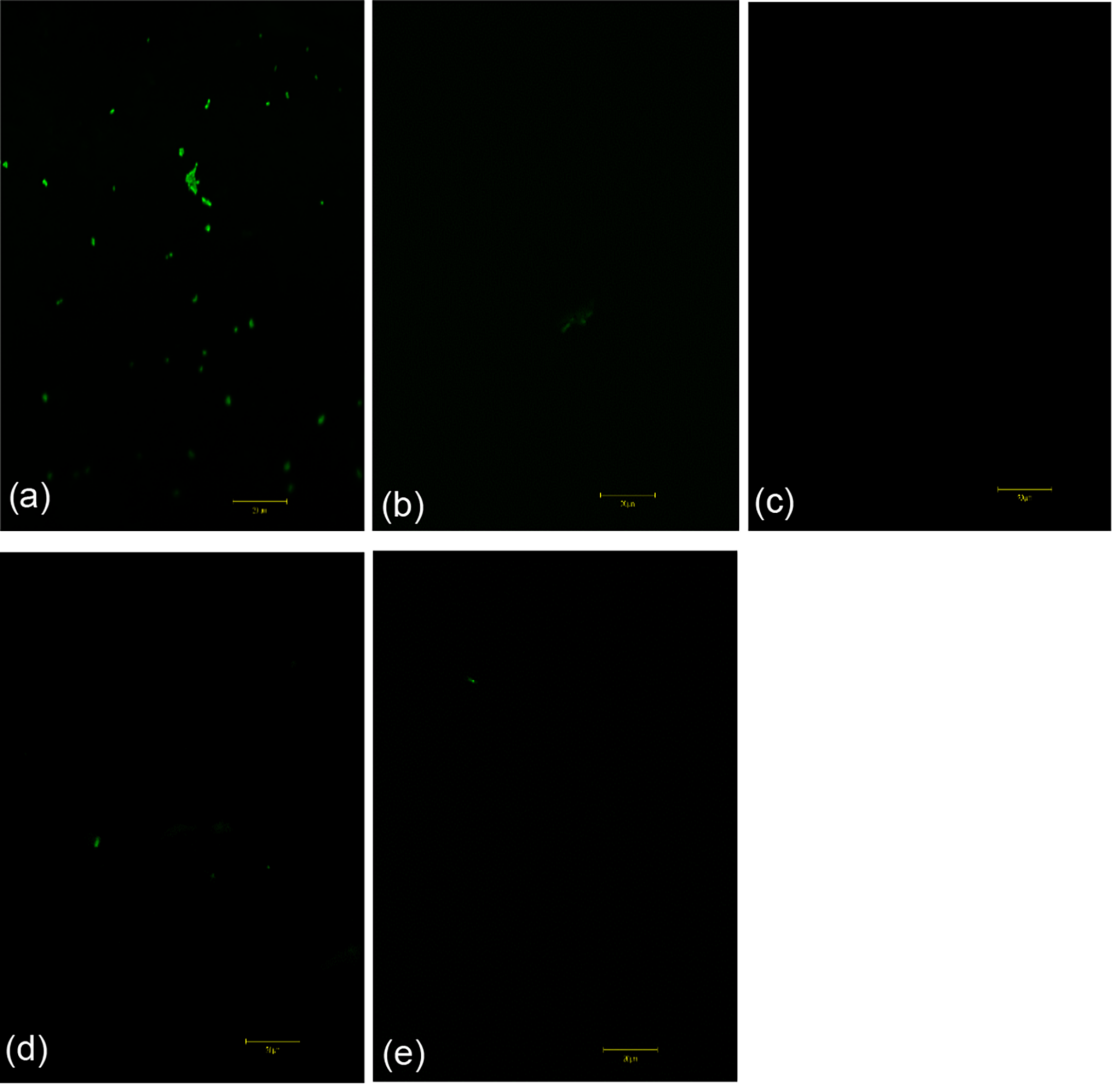
Confocal laser scanning micrograph of **a** DMSO **b** azithromycin **c** mangiferin **d** ZINC D **e** ZINC E treated cells (magnification x63)

Since biofilm development is a significant virulence factor of *Las* QS, the effects of testing the AQS effect on it involve visualizing structural density as an indicator of the architectural viability once the lead compounds have been added. This was visualized under confocal laser microscope (Rolland et al. 2006) where stained biomass with adherent biofilm development is visibly quantified by fluorescence in relation to the amount of present biofilm development. The negative control (Fig. 9a) was DMSO, which saw bacterial growth and biofilm development as indicated by the bright green fluorescence of several scattered clusters with high bacterial biofilm viability. This was compared with treatment with the reference standard azithromycin (Fig. 9b) and the 3 lead compounds (Fig. 9c – mangiferin, Fig. 9d – ZINC D, Fig. 9e – ZINC E) at MIC. At this concentration, all compounds may allow bacterial growth to occur but reduce overall biofilm development.

The reference standard was shown to successfully inhibit biofilm formation tremendously as only faint fluorescence was visible. This is reflective of reduced cell attachment and corresponds with a similar observation that reported azithromycin to inhibit and impair extracellular matrices when tested against *P. aeruginosa* (Kumar et al. 2021).

Compared to the reference standard, ZINC D and E both showed similarly reduced biofilm expression in the bacteria. A single, distinct spot of aggregation is represented by the brighter fluorescence shown for each compound than the reference standard. This suggests ZINC D and E to have weaker inhibition than azithromycin which was preliminary suggested in the cell attachment assay. However, this sparser distribution of cells in the control still shows a biofilm matrix present, more faint and not as dense as the remnants of the ZINC D and E tests, but certainly more prevalent as supported by the assay for biofilm development inhibition.

Of the 3 lead compounds, mangiferin maintained the observation of high cell attachment inhibition as well as high biofilm inhibition. This is evident by the mangiferin test showing virtually no fluorescence, indicating no biofilm development, possibly due to near-eradicated cell adherence. This is a successful reflection of *LasR-*QS inhibition more so than ZINC D, E, and the reference standard itself. The effectiveness of mangiferin on biofilm development has been reported previously when tested against *E. coli* (Prabhu et al. 2023). The AQS effect of the 3 lead compounds has been effectively communicated but reveals mangiferin to be the most inhibitory compound of biofilm development as well as cell attachment over 24 h.

#### Pyocyanin production inhibition

Pyocyanin is a blue-green molecule responsible for the morphological green hue characteristic of *P. aeruginosa* and is heavily implicated in the pathogen’s virulence strategy as a cytotoxic secondary metabolite. As a QS-mediated virulence factor, the levels of its production inhibition were tested.

**Fig. 10:**
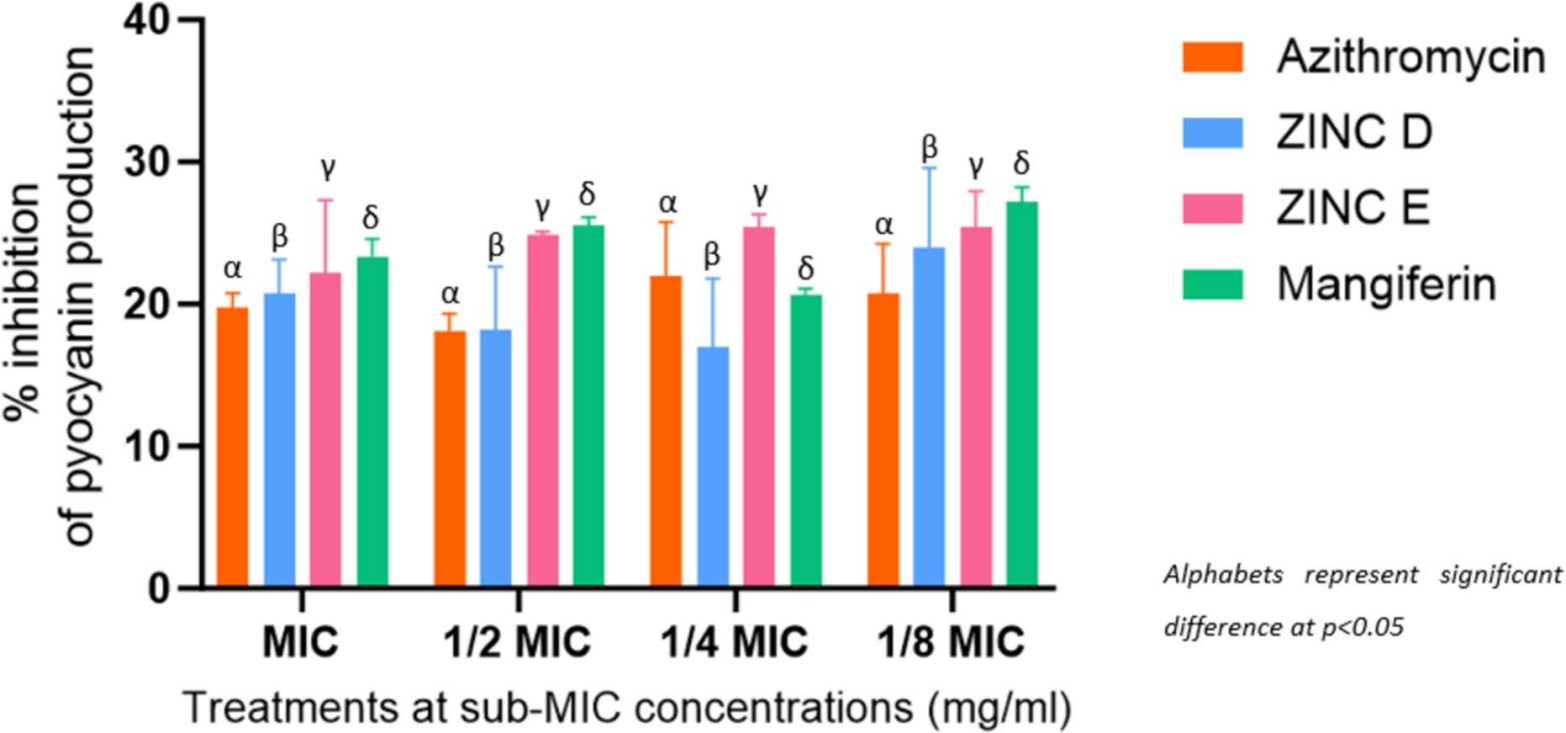
Inhibitory effect of test compounds on pyocyanin production, a toxin virulence factor regulated by QS at sub-MIC concentrations (mg/ml)

Rather surprisingly, neither the 3 lead compounds nor the reference standard inhibited production of the toxin beyond 30%. This observation contradicts reports of higher reductions (70.48%) by Aleanizy et al. (2021) and (89%) by Husain et al. (2017) which used methanol-extraction of *M. indica* on *P. aeruginosa* and reported a relatively consistent level of inhibition. However, the results in this study concur with similarly low levels reported by Elshaer and Shabaan (2021). With this observation in mind, the compounds may not have demonstrated adequately high levels of QS inhibition in the context of pyocyanin production, but show relatively consistent inhibition at all concentrations, with ZINC E and mangiferin proving to be better QSI agents than the reference standard, especially at lower concentrations (Fig. 10).

These compounds could be further optimized for improved levels of inhibition, especially as pyocyanin regulation may have been affected by *Rhl* behaving as a surrogate in place of non-functional *LasR* as reported by de Oliveira Pereira et al. (2023), which contradicts O’Loughlin et al. (2013) mentioning that non-functionality (by deletion) of *LasR* affects associated gene expression, including *Rhl.* Despite the significance of pyocyanin production as an anti-virulence target (Morkunas et al. 2016), the exploration of other QS-mediated toxins is encouraged. This study serves as an indicator of the 3 lead compounds’ AQS activity comparable to the reference standard in terms of toxin production inhibition, furthering their stance as QSI agents.

#### Motility assays

Motility within *P. aeruginosa* is the result of QS-regulated locomotion of type IV pili (Yeung et al. 2009), resulting in a turbid zone of cellular branching on semi-solid media surfaces. Both *Las* and *rhl* regulate swarming motility and play roles in the propagation and intercellular signaling in response to stimuli. QSI would aid in mitigating motility and thus bacterial surface area coverage for proliferation, which was point of this study’s investigation. As was reported by Hussain et al. (2017) *M. indica’s* primary phytochemical, pyrogallol, reported a concentration-dependent effect on QS in terms of motility. This was similar to this investigation, which noted that as mangiferin’s concentration decreased, zone diameter increased for bacterial swarming-swimming. The same trend was observed for the reference standard, although at the lowest tested concentration, mangiferin yielded smaller-sized zones. For swarming, ZINC D and E showed the highest inhibition of all compounds at 1/8 MIC, but at MIC for swimming motility (Table 7). From this study, the data correlates with the discoveries of Kapadia et al. (2022) stating that with inhibited swarming motility comes inhibited QS phenotypes, as is suggested by the smaller swarming zones than the control. While swimming falls (Table 8) more so under the regulation of *Rhl* (Li et al. 2014), sufficient *Las* inhibition should reflect in this QS system’s expression, which was the case, albeit not to the same degree as mentioned by Xie et al. (2022) reporting zone diameters significantly lower (1.47 ± 0.12 cm) when testing a flavone derivative.

**Table 7:** Inhibitory effect of test compounds on QS-mediated swarming motility in *P. Aeruginosa* at sub-MIC concentrations (mg/ml)

Zone diameters of test compounds (mm)				
Concentration	Mangiferin	ZINC D	ZINC E	Azithromycin (control)
MIC	4.50	5.00	4.83	8.33
½ MIC	5.83	5.17	4.67	4.66
¼ MIC	11.50	11.50	12.17	4.50
1/8 MIC	6.50	6.50	5.00	13.50

**Table 8:** Inhibitory effect of test compounds on QS-mediated swimming motility in *P. Aeruginosa* at sub-MIC concentrations (mg/ml)

Zone diameters of test compounds (mm)				
Concentration	Mangiferin	ZINC D	ZINC E	Azithromycin (control)
MIC	20.0	18.6	13.0	17.0
½ MIC	17.3	28.3	20.0	12.0
¼ MIC	26.6	29.0	22.6	19.0
1/8 MIC	33.3	23.6	13.6	37.7

## Conclusion

A library of compounds related to mangiferin was investigated in this study for their binding interactions and stability towards *P. aeruginosa LasR. LasR* facilitating the severe pathogenicity of *P. aeruginosa* as it forms part of its QS system contributing significantly to the MDR status of the organism (Pattnaik et al. 2018). In this study, mangiferin and its related compounds assessed the possibility of reducing transcription and expression of virulence factors mediated by QS including biofilm formation which may occur with the inhibition of the protein. The data obtained revealed the resulting complexes of ZINC00821593 (ZINC E) and ZINC00335421 (ZINC D) with *LasR* having higher negative binding free energy compared to mangiferin-*LasR* and azithromycin-*LasR*. This observation was rooted in the capability of the putative leads to form compact and stable *LasR* complexes. Further insight into the in vitro analyses showed that at sub-minimum inhibitory concentrations, ZINC D exhibiting adequate to significant levels of *LasR* inhibition through reduced QS-mediated phenotype expression than azithromycin and quantitatively competed favourably with mangiferin. Since the data obtained from the computational models harmonized with the in vitro result, it might mean that mangiferin and ZINC D are capable of mitigating *P. aeruginosa* infections by inhibiting the *LasR* protein to reduce virulence factor transcription such as cell attachment, biofilm development, swarming and swimming motility and even pyocyanin production more so than the reference standard. Mangiferin and ZINC D could therefore be further investigated for their anti-QS abilities through interference with autoinducer production, destruction of autoinducer molecules, synergistic effects, or inhibition of QS in other microbial circuits and may be potential candidates for novel drugs in the course of action against *P. aeruginosa* infections.

## Supplementary Information

Below is the link to the electronic supplementary material.

Supplementary Material 1 (DOCX 1542 KB)

## Data Availability

No datasets were generated or analysed during the current study.
