# Supplementary material for: Molecular modelling and experimental validation of mangiferin and its related compounds as quorum sensing modulators of Pseudomonas aeruginosa
Source: Arch Microbiol. 2025 Feb 8;207(3):53. doi: 10.1007/s00203-025-04240-3 (PMC11807064; doi:10.1007/s00203-025-04240-3)
Supplement: Supplementary file 1 — Supplementary Material 1 (DOCX 1542 KB) [file 203_2025_4240_MOESM1_ESM.docx]

**SUPPLEMENTARY MATERIALS**

**Molecular modelling and experimental validation of mangiferin and its related compounds as anti-quorum sensing therapeutics against *Pseudomonas aeruginosa***

**Table S1:** Docking scores from AutoDock Vina for all 133 screened compounds from ZINCPharmer

| ZINC ID | Docking score (kcal/mol) |
| --- | --- |
| ZINC00027860 | -11.6 |
| ZINC00047398 | -10.5 |
| ZINC00047577 | -12.2 |
| ZINC00058417 | -10.9 |
| ZINC00058418 | -9.6 |
| ZINC00058420 | -11.1 |
| ZINC00078102 | -8.2 |
| ZINC00081942 | -10.3 |
| ZINC00089623 | -11.0 |
| ZINC00106135 | -6.0 |
| ZINC00107237 | -9.3 |
| ZINC00107243 | -8.6 |
| ZINC00107246 | -8.4 |
| ZINC00111053 | -10.7 |
| ZINC00117011 | -10.6 |
| ZINC00117080 | -6.9 |
| ZINC00117107 | -10.6 |
| ZINC00117109 | -10.5 |
| ZINC00117112 | -10.6 |
| ZINC00117115 | -10.4 |
| ZINC00159983 | -9.1 |
| ZINC00172333 | -8.3 |
| ZINC00172335 | -9.7 |
| ZINC00174951 | -11.2 |
| ZINC00175284 | -5.7 |
| ZINC00180363 | -10.7 |
| ZINC00187249 | -10.5 |
| ZINC00189067 | -9.3 |
| ZINC00189074 | -9.3 |
| ZINC00189085 | -9.1 |
| ZINC00192940 | -12.5 |
| ZINC00192967 | -10.5 |
| ZINC00192976 | -12.0 |
| ZINC00197621 | -12.4 |
| ZINC00209718 | -9.9 |
| ZINC00213206 | -9.2 |
| ZINC00213209 | -8.4 |
| ZINC00213210 | -8.6 |
| ZINC00213212 | -7.9 |
| ZINC00213214 | -6.8 |
| ZINC00215026 | -9.4 |
| ZINC00215028 | -9.0 |
| ZINC00215030 | -9.2 |
| ZINC00241135 | -11.2 |
| ZINC00259918 | -11.4 |
| ZINC00273028 | -9.7 |
| ZINC00298186 | -8.4 |
| ZINC00333687 | -10.8 |
| ZINC00335421 | -11.7 |
| ZINC00336477 | -12.6 |
| ZINC00352330 | -9.3 |
| ZINC00352337 | -7.6 |
| ZINC00369431 | -7.2 |
| ZINC00390824 | -7.5 |
| ZINC00392088 | -7.8 |
| ZINC00392089 | -8.1 |
| ZINC00406742 | -7.7 |
| ZINC00439500 | -9.8 |
| ZINC00500352 | -5.8 |
| ZINC00546546 | -11.0 |
| ZINC00562023 | -8.5 |
| ZINC00568064 | -6.1 |
| ZINC00614532 | -10.5 |
| ZINC00616524 | -10.4 |
| ZINC00620537 | -6.9 |
| ZINC00621243 | -9.6 |
| ZINC00625966 | -8.9 |
| ZINC00637920 | -7.6 |
| ZINC00666210 | -10.1 |
| ZINC00670049 | -10.0 |
| ZINC00672082 | -10.9 |
| ZINC00702142 | -6.1 |
| ZINC00709214 | -5.8 |
| ZINC00756962 | -5.8 |
| ZINC00779685 | -6.9 |
| ZINC00779823 | -6.5 |
| ZINC00800893 | -9.7 |
| ZINC00811862 | -11.8 |
| ZINC00821592 | -9.7 |
| ZINC00821593 | -11.6 |
| ZINC00821845 | -10.8 |
| ZINC00825226 | -8.5 |
| ZINC02863600 | -5.1 |
| ZINC02863602 | -6.4 |
| ZINC02863603 | -5.8 |
| ZINC02863606 | -7.7 |
| ZINC02863607 | -7.1 |
| ZINC04905896 | -11.3 |
| ZINC06759201 | -5.8 |
| ZINC06759202 | -6.9 |
| ZINC06759913 | -6.1 |
| ZINC06760086 | -7.4 |
| ZINC06760677 | -5.7 |
| ZINC06760678 | -5.9 |
| ZINC08591032 | -6.9 |
| ZINC08591035 | -6.8 |
| ZINC08591070 | -6.2 |
| ZINC08591072 | -5.8 |
| ZINC08591079 | -3.5 |
| ZINC08591082 | -5.4 |
| ZINC09302964 | -5.5 |
| ZINC09304942 | -7.7 |
| ZINC09305185 | -5.5 |
| ZINC09305186 | -6.5 |
| ZINC09410404 | -5.2 |
| ZINC09410753 | -5.3 |
| ZINC09418401 | -6.1 |
| ZINC09418402 | -6.3 |
| ZINC09419783 | -6.1 |
| ZINC09435310 | -6.7 |
| ZINC09435570 | -5.5 |
| ZINC09435749 | -1.5 |

**Table S2:** Plots of interactions of the top-ranked metabolites and reference standard towards *LasR* at different time intervals during the 140 ns MD simulation

| COMPLEX | DURATION | 2D INTERACTTION |
| --- | --- | --- |
| *LasR* + mangiferin | 50 | 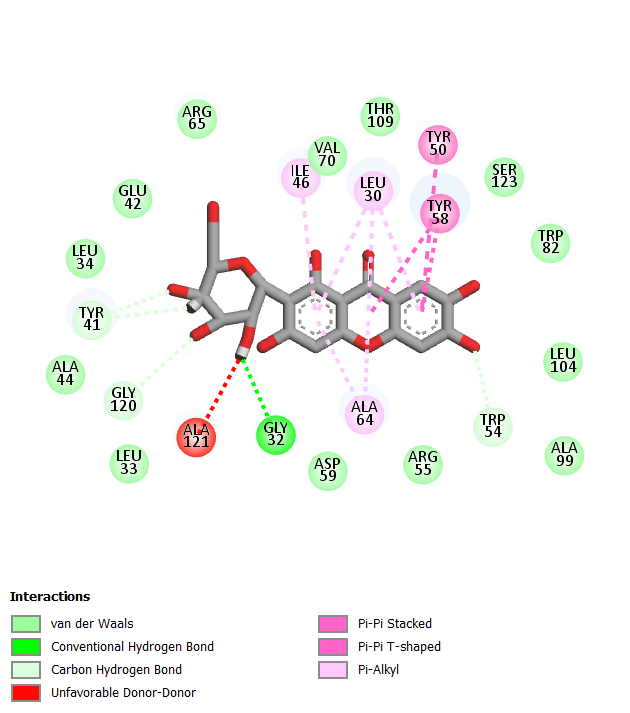 |
|  | 100 | 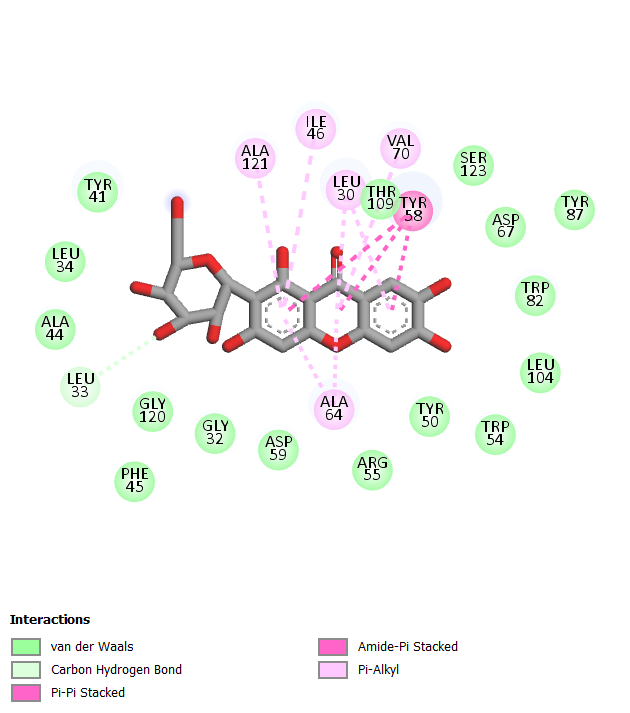 |
|  | 140 | 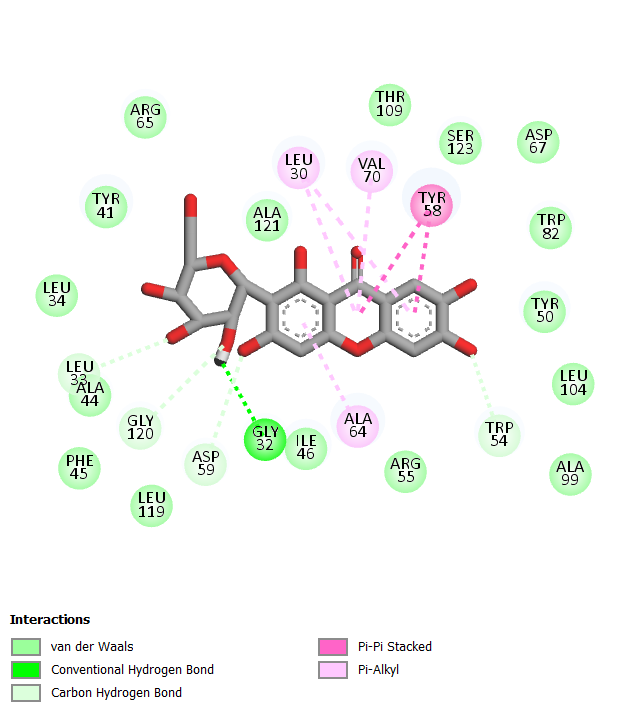 |
| *LasR* + Azithromycin | 50 ns | 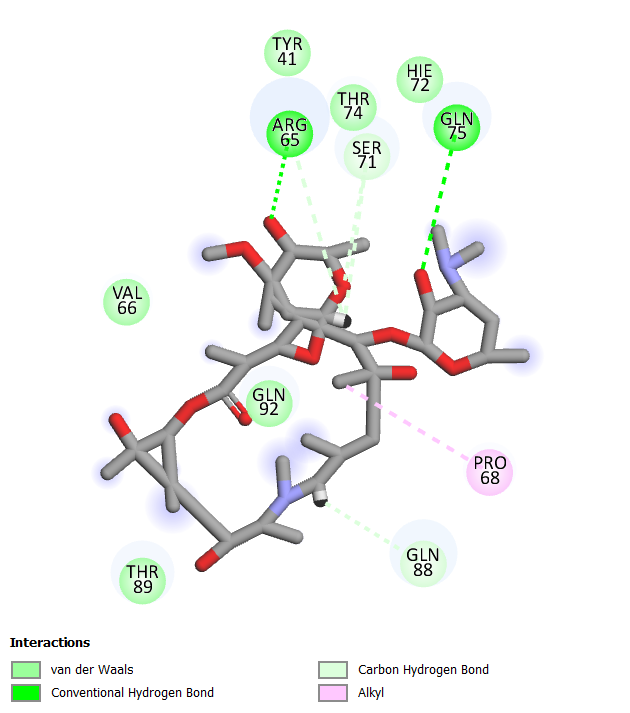 |
|  | 100 ns | 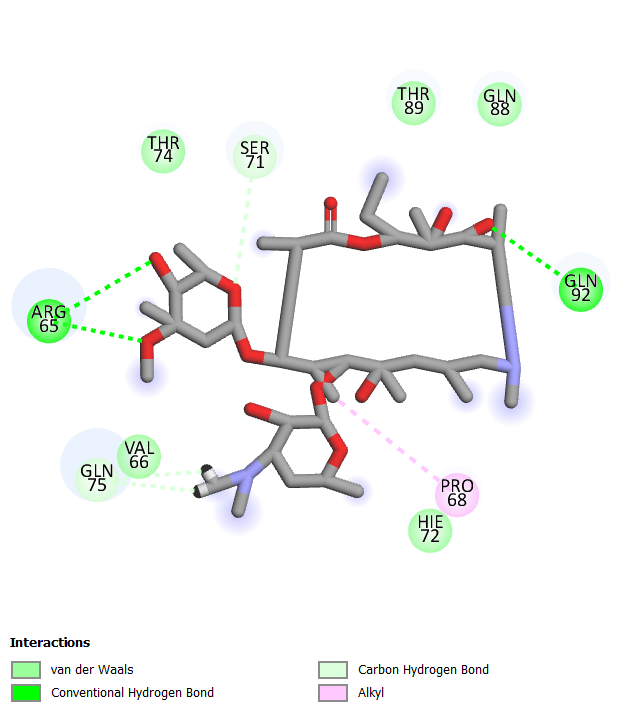 |
|  | 140 ns | 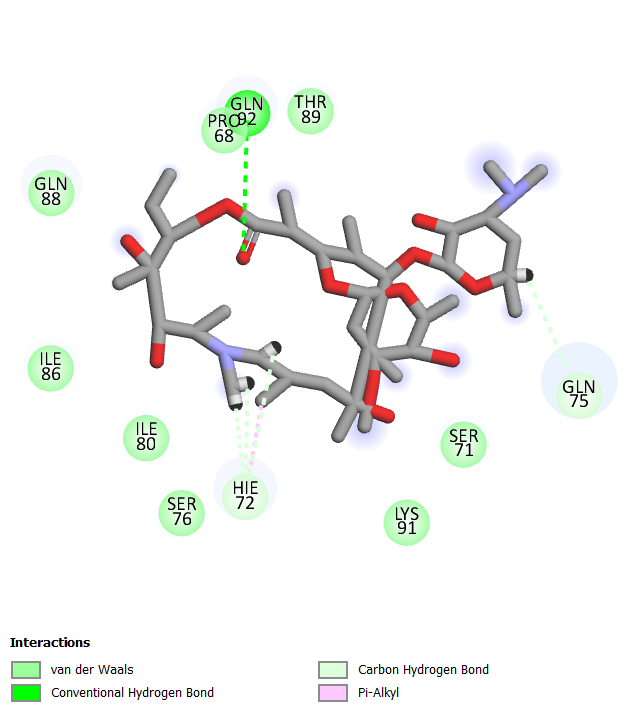 |
| *LasR* + ZINC00117011 | 50 ns | 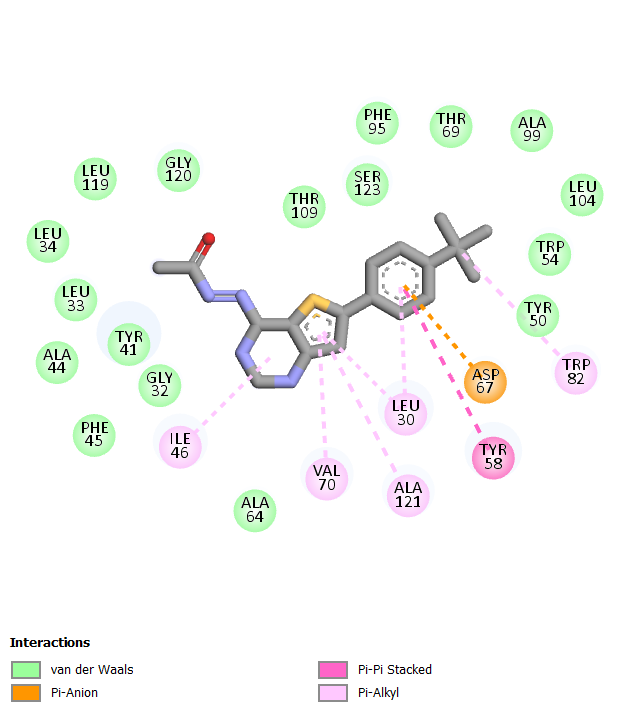 |
|  | 100 ns | 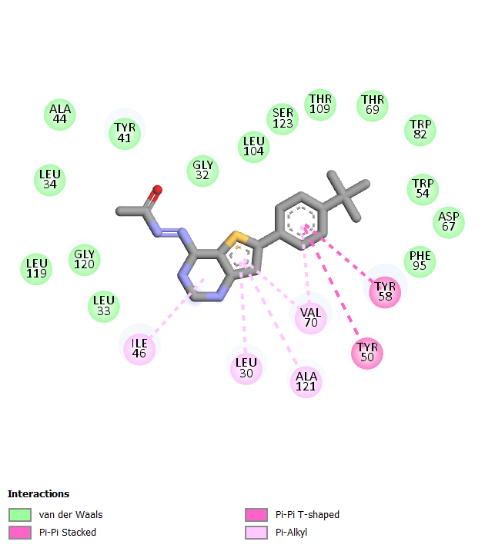 |
|  | 140 ns | 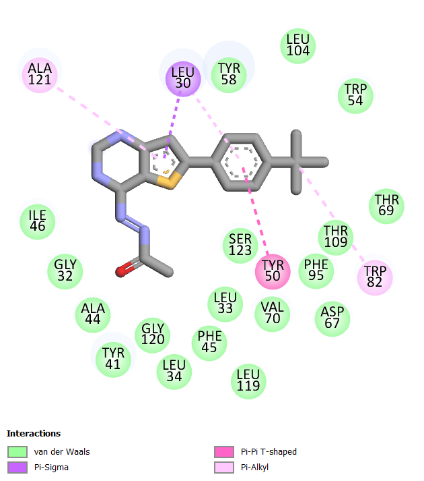 |
| *LasR* + ZINC00117107 | 50 ns | 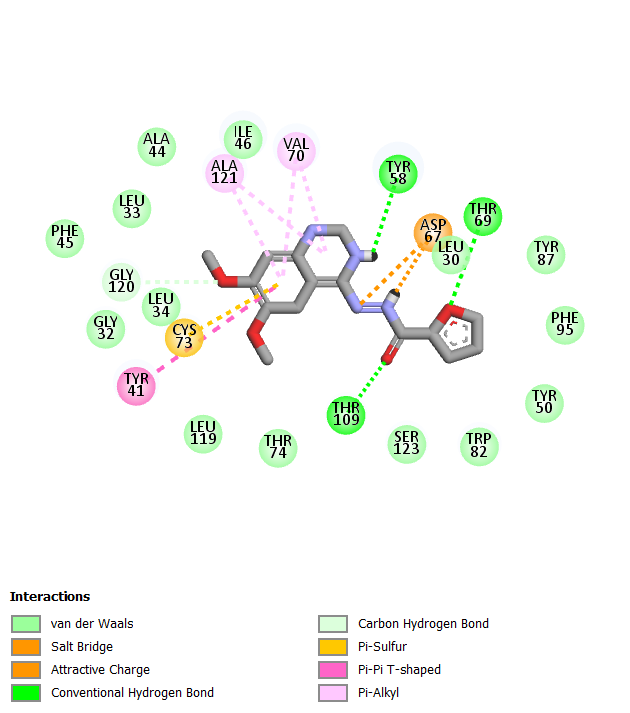 |
|  | 100 ns | 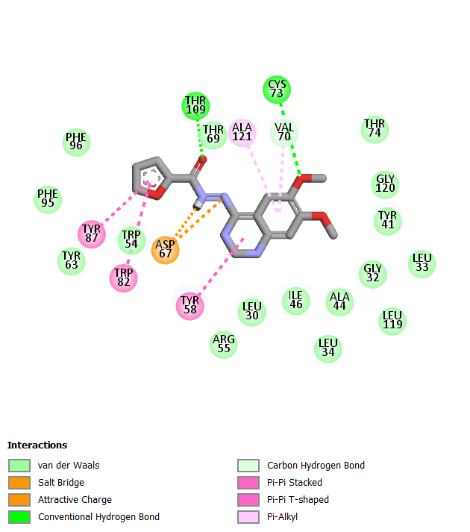 |
|  | 140 ns | 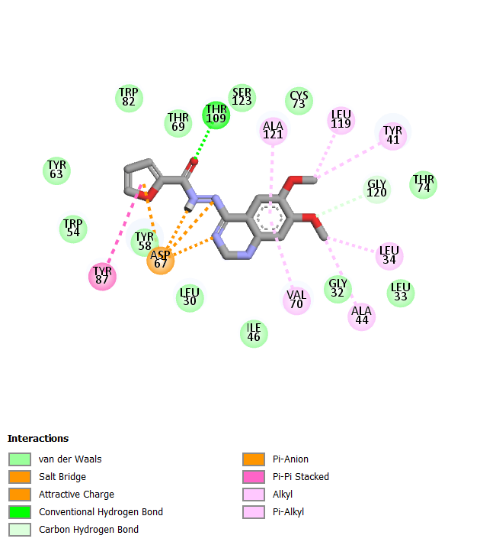 |
| *LasR* + ZINC00333687 | 50 ns | 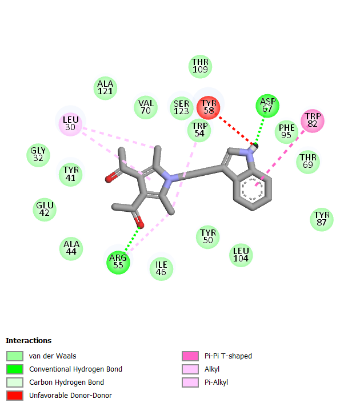 |
|  | 100 ns | 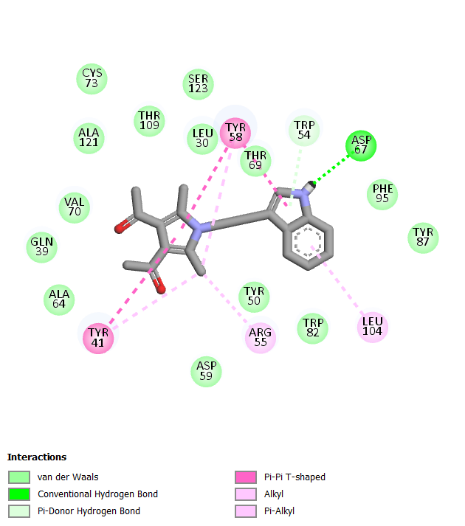 |
|  | 140 ns | 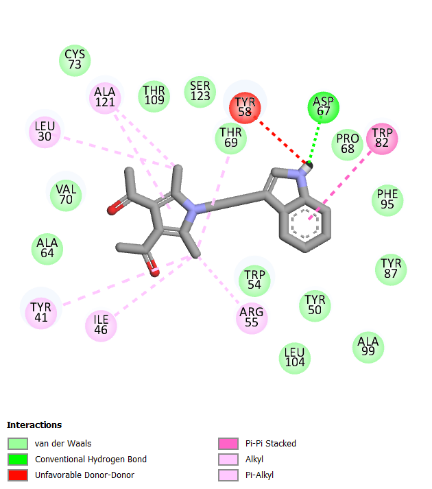 |
| *LasR* + ZINC00335421 | 50 ns | 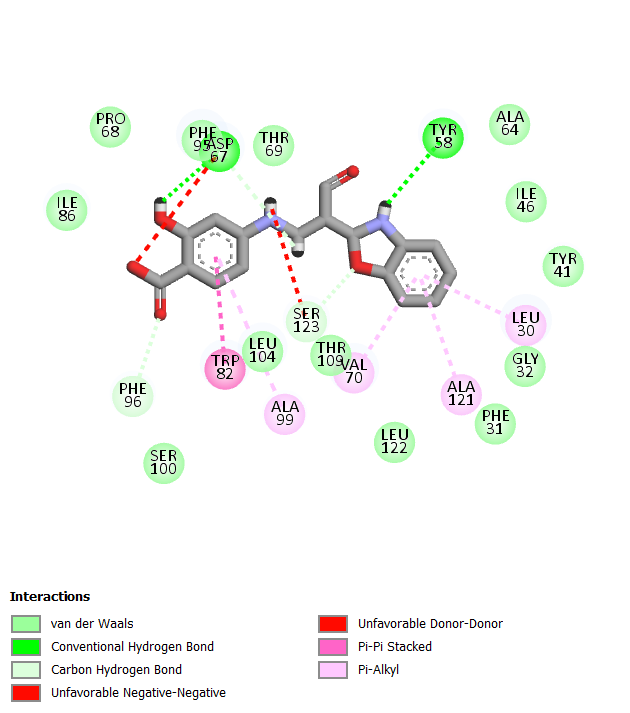 |
|  | 100 ns | 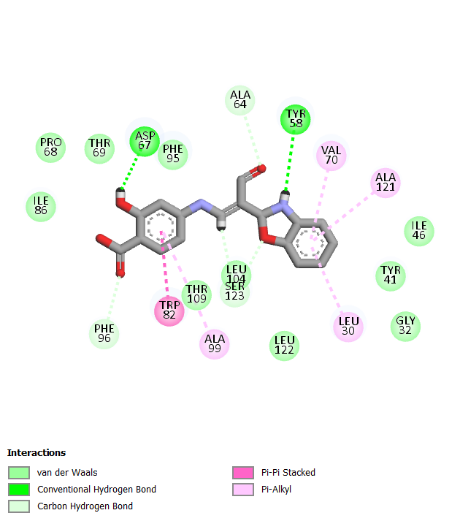 |
|  | 140 ns | 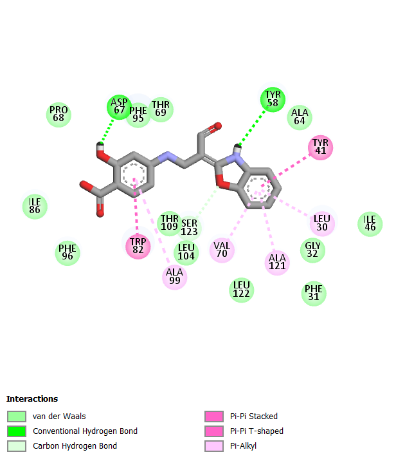 |
| *LasR* + ZINC00821593 | 50 ns | 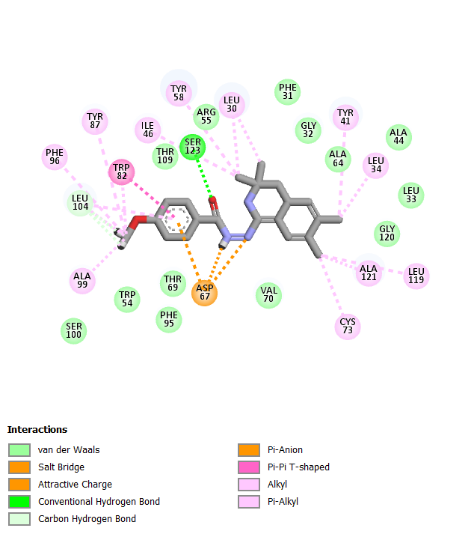 |
|  | 100 ns | 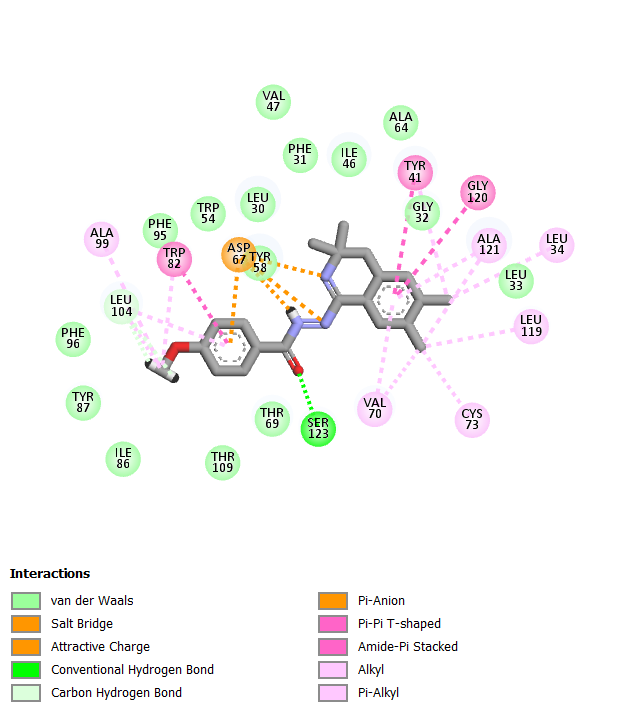 |
|  | 140 ns | 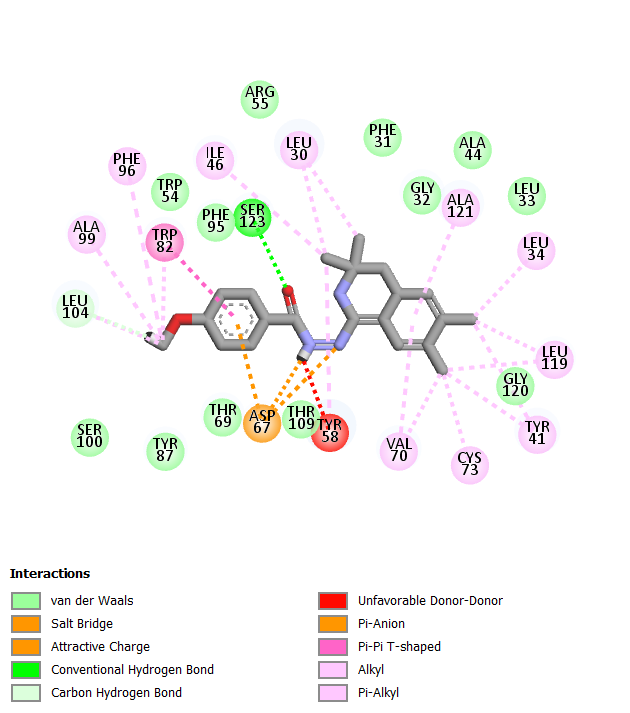 |
